# PHO 2019 Conference Abstracts: San Diego, CA, USA. 25-26 October 2019

**DOI:** 10.1186/s41687-019-0157-7

**Published:** 2019-11-26

## P1. Towards developing mobility ontology in Acquired Brain Injury (ABI) population: an umbrella review of mobility PROMs

### Rehab Alhasani^1,2,6^, Cluadine Auger^2,4^, Sara Ahmed^1,2.3,5^

#### ^1^School of Physical and Occupation Therapy, McGill University, Montreal, Canada; ^2^Centre de Recherche Interdisciplinaire en Réadaptation (CRIR), Montreal, Canada; ^3^Constance Lethbridge Rehabilitation Center, Montreal, Canada; ^4^Université de Montréal, School of Rehabilitation, Montreal, Canada; ^5^Centre de réadaptation Lucie-Bruneau du Centre integré universitaire de sante et de services sociaux (CIUSSS) du Centre-Sud-de-l’Ile-de-Montréal, Montreal, Canada; ^6^Princess Noura Bint Abdulrahman University, Riyadh, Saudi Arabia

##### **Correspondence:** Rehab Alhasani (rehab.alhasani@mail.mcgill.ca)

**Background**

Our group is developing a patient portal as part of digital infrastructure to systematically collect PROMIS measures and clinical data to support decision making in rehabilitation care. To tailor mobility interventions to patient sub-groups, mobility ontology is needed to link data from multiple sources (PROMs, clinician, technology). As a first step to develop the ontology, an umbrella review was conducted to identify mobility PROMs; and to map the mobility domains from PROMs to the International Classification of Functioning, Disability and Health Framework (ICF)1 and the Webber’s framework.2 A secondary objective was to map the extent to which the PROMIS mobility item bank covered each of the identified domains.

**Methods**

MEDLINE, CINAHL, Cochrane and EMBASE were systematically searched for systematic reviews of mobility measures for the ABI population. Two investigators independently screened abstracts and full texts against pre-defined criteria and extracted data. References of included systematic reviews were hand-searched. Mobility measures, including their domains, from each systematic review were mapped to the ICF and Webber’s framework. PROMIS mobility items were mapped to items identified in the literature.

**Results**

Among 9 systematic reviews, 215 mobility items across 39 mobility PROMs were identified in the ABI population. Based on the ICF, mobility items were categorized at the level of body function (13%), activity (67%), participation (10%) and environmental factors (6%). According to Webber’s framework, factors influencing mobility were covered across physical (45%), psychosocial (21%), cognition (10%), and environmental (19%). None of the measures covered the personal factors. Although PROMIS covered most of the items in the extracted mobility PROMs, none of the systematic reviews included PROMIS mobility.

**Conclusions**

Mobility PROMs covered most of the relevant items in the ICF and Webber’s framework. Reviews did not include PROMIS mobility measure and this may be because it has not been tested in the ABI population. Mobility PROMs used different terminology to describe the same domain and perationalized items and measurement scales covering the same content differently. Thus, developing mobility ontology will provide a common language and allow mapping between relevant mobility items, making it easier to map data across multiple sources to evaluate mobility and conduct comparative effectiveness of rehabilitation interventions.

## P2. Differences in reported pain among patients with low back pain: PROMIS-10, NRS, and ODI

### Mark Alan Fontana^1,2^, Catherine H. MacLean^1^, Harvinder S. Sandhu^1^, Sheeraz Qureshi^1^, Vinicius C. Antao^1^

#### ^1^Center for the Advancement of Value in Musculoskeletal Care, Hospital for Special Surgery, New York, NY, USA; ^2^Department of Healthcare Policy and Research, Weill Cornell Medical College, New York, NY, USA

##### **Correspondence:** Vinicius C. Antao (AntaoV@hss.edu)

**Objective**

To compare pain scores as measured by single questions from three instruments administered the same day to patients with lumbar spine disease.

**Methods**

Responses to a numeric pain rating scale (NRS, 0–10 scale), and single pain items on each the PROMIS Scale v1.2 – Global Health (PROMIS-10) (0–10 scale) and the Oswestry Disability Index (ODI, 6 options) were compared among patients presenting to one of 15 spine surgeons at a single facility between January 2017 and April 2019. The PROMIS-10 and NRS could be directly compared given their identical scales. To compare either the PROMIS-10 pain item or NRS to the ODI pain item, we collapsed the 0–10 scales to 6 options by consolidating responses to maximize agreement between each pair of instruments. For each pair of surveys, we report the Spearman correlation coefficient between responses, as well as the percentages of responses that identically match, that are off by one point, and are off by more than one point.

**Results**

Among 25,497 total patients, there were 5,084 with responses to 2/3 of the survey questions on the same day, and 2,777 with responses to all three questions on the same day. For the 2,981 patients with responses to both the PROMIS-10 and the NRS, the correlation was 82%; 57% answered identically between the two instruments; 84% answered within one point of the other; and 16% answered two or more points differently. Comparing the collapsed PROMIS-10 to the ODI, there were 7,168 patients with responses to both; the correlation was 75%; 55% answered identically between the two instruments; 93% answered within one point of the other; and 7% answered two or more points differently. For the collapsed NRS and ODI, there were 3,075 patients with responses to both; the correlation was 68%; 52% answered identically; 92% answered within one point of the other; and 8% answered two or more points differently.

**Conclusions**

Patients with lumbar spine disease report similar levels of pain according to the NRS and single pain items on the PROMIS-10 and ODI.

## P3. Assessing use of PROMIS outcomes in pediatric neuromuscular scoliosis patients

### Liam Wong^1^, Reed Ling^1^, Madeleine A.Z. Ball^1^, Yashar Javidan^1,2^, Eric O. Klineberg^1,2^ , Rolando F. Roberto^1,2^

#### ^1^Shriners Hospitals for Children Northern California, Sacramento, CA, USA; ^2^University of California Davis Orthopaedic Surgery, Sacramento, CA, USA

##### **Correspondence:** Liam Wong (lwonglwong@gmail.com)

**Background**

Neuromuscular scoliosis (NS) is primarily characterized by progressive spinal curvature due to Duchenne muscular dystrophy (DMD), cerebral palsy (CP), and spina bifida (SB). These patients possess a broad range of physical function, pain levels, and communication abilities. To assess and validate use of PROMIS in NS, we will differentiate between parent-reported proxy and self-report (SR) for normative Pain Interference (Pain), Upper Extremity Function (UE), Peer Relationships (Peer), and Mobility scores. Our goal is to elucidate significant score differences by severity levels assessed by Gross Motor Function Classification Scale (GMFCS 1-5), major NS diagnoses (DMD, SB, CP), and reported pain (mild-normal, moderate-severe).

**Methods**

In this IRB-approved single-center retrospective review, we analyzed NS PROMIS scores for 615 children aged 5-17 between July 28, 2017 and March 11, 2019. Raw scores were converted to t-scores with a mean of 50 and a standard deviation 10. Student t-tests identified differences between groups.

**Results**

Pain and Peer mean scores were in normal range for 357 SR and 259 proxy subjects, but depressed in Mobility and UE. Significant differences (p<0.00005) were found between SR and proxy for all tested domains. When stratifying by GMFCS (1-2 vs. 3-5), subjects with increased severity had lower mean Mobility (27.87) and UE (18.90) (p<2.16E-41). CP subjects (n=491) had significant disagreement between SR and proxy (p<3.96E-5); DMD (n=15) had the highest reported mean pain score (54.62). Subjects with normal pain had reduced Mobility and UE; both domains were significantly lower (p<1.56E-13) for subjects with pain.

**Conclusions**

NS children report impacted Mobility and UE scores. When parents answer for their children, they report higher Pain and lower UE, Mobility, and Peer scores. Elevated GMFCS (3-5) and Pain had the greatest correlation to lower Mobility and UE scores. Results show significant differences between SR and proxy by NS severity level, diagnosis, and pain scores establishing the need for further investigation into the use of proxy and SR methods.

## O4. Using PROMIS to determine if the patient acceptable symptom state differs by socioeconomic status

### David N. Bernstein^1^, Kiah Mayo^1^, Judith F. Baumhauer^1^, Chris Dasilva^1^, Kathleen Fear^1^, Jeff R. Houck^2^

#### ^1^University of Rochester Medical Center, Rochester, New York, USA; ^2^George Fox University, Newberg, Oregon, USA

##### **Correspondence:** David N. Bernstein (David_Bernstein@URMC.Rochester.edu)

**Background**

Understanding the impact socioeconomic status (SES) plays on patient-reported outcome measures (PROMs), such as PROMIS, and patient satisfaction is crucial to ensure health equity. We sought to determine whether SES factors impact the patient acceptable symptom state (PASS) threshold and PROMIS scores in an orthopaedic foot and ankle population.

**Methods**

Between 2/15-12/17, foot and ankle patients presenting for new patient visits to an academic clinic completed PROMIS PF, PI, and Depression, as well as answered the PASS question. SES factors (e.g., age, sex, race, ethnicity) were recorded from patient charts and using Census Block Groups (CBGs). Chi-square two-way ANOVA with pairwise comparisons, and receiver operating characteristic (ROC) curve analyses were used to evaluate the impact of SES factors on PROMIS scores and PASS status.

**Results**

A total of 2,597 patients were analyzed. While small, age was the only patient factor that was associated with a difference in PASS rate (15% vs. 11%). For PROMIS PF, PI, and Depression, the average difference between patients in the highest lowest income brackets was 4.6, -5.8, and -5.0, respectively. The PROMIS PF PASS threshold for the highest income bracket was near the population mean (48.9), while the PROMIS PF PASS threshold for the lowest income bracket was more than a standard deviation below the population mean (39.4). Similarly, the PROMIS PI PASS threshold differed by 5.7 points when comparing the lowest and highest income brackets. PROMIS Depression was unable to discriminate PASS status.

**Conclusions**

Patients in the lowest income bracket reported significantly worse symptoms and perceived them as satisfactory, while the opposite occurred for patients in the highest income bracket. Possible explanations for this discrepancy include unequal access to care and inflated expectations of healthcare outcomes based on SES factors. This raises important ethical questions focused around autonomy, justice, beneficence, and non-maleficence.

## P5. Early improvement in physical function after symptomatic syndesmotic screw removal

### Jessica M. Kohring, Catherine A. Humphrey, Kyle T. Judd, Gillian Soles, John T. Gorczyca, John P. Ketz, Judith F. Baumhauer

#### University of Rochester Medical Center, Rochester, NY, USA

##### **Correspondence:** Judith F. Baumhauer (judy_baumhauer@urmc.rochester.edu)

**Background**

There is questionable need for hardware removal after ankle fracture fixation and surgeon variation in performing this surgery. The purpose of this study was to investigate the early impact of syndesmotic screw removal on PROMIS outcomes and ankle range of motion (ROM) in patients who had ankle fracture with syndemosis screw placement.

**Methods**

58 ankle fractures with syndesmotic injury that required ORIF with syndesmotic fixation and subsequent had painful syndesmotic screw symptoms and had subsequent removal met criteria for inclusion from February 2015 to May 2018. We analyzed PROMIS scores collected just prior to syndesmotic screw removal and at the first post-operative visit. A retrospective chart review was performed to collect demographic and ankle ROM data. Cohort data was collected for 71 patients who underwent ORIF with syndesmotic fixation but had no screw symptoms and did not have screw removal during the same study period.

**Results**

The PROMIS physical function (PF) T-score was 35.2 at an average of 106 days after ORIF just prior to syndesmotic screw removal. There was a statistically significant improvement in the PF T-score to 44.5 (p<0.01) in the immediate post-operative period after screw removal. There was statistically significant improvement in ankle ROM after screw removal (p<0.01). In a cohort comparison group of 71 patients during the same time period who did not undergo syndesmotic screw removal, the PF T-score was 41.6 at a mean 150 days after surgery, similar to the PF T-score (44.5) for patients after syndesmotic screw removal (p=0.06), Table 1.

**Conclusions**

In our study, there was an immediate clinically meaningful improvement in physical function outcomes and ankle ROM after symptomatic syndesmotic screw removal for patients who underwent ankle fracture ORIF with syndesmotic fixation, similar to asymptomatic patients who did not require syndesmotic screw removal within the same post-operative timeframe. This provided strong evidence that patient will benefit from symptomatic screw removal and it did change the care provided to these trauma patients.

## P6. How well do patients recover compared to population norms after an ankle arthrodesis surgery?

### Judith F. Baumhauer^1^, Jessica M. Kohring^1^, Irvin Oh^1^, Sam Flemister^1^, John P. Ketz^1^, Jeffrey R. Houck^2^

#### ^1^University of Rochester Medical Center, Rochester, NY, USA; ^2^George Fox University, Newberg, OR, USA

##### **Correspondence:** Judith F. Baumhauer (judy_baumhauer@urmc.rochester.edu)

**Background**

Generic patient reported outcomes (PRO’s) after surgery typically do not compare patient status to expected normative data. Ankle arthrodesis is an end stage procedure that may permanently impair ankle function however relieves pain. Understanding the extent that ankle arthrodesis restores overall physical function(PF), pain interference(PI) and depression relative to population norms will assist with provider/patient decisions to have the surgery and recovery after.

**Objective**

The objective was to determine how Patient Reported Outcome Measurement Information (PROMIS) PI, PF, and Depression scales pre-operatively and postoperatively compare to population norms.

**Methods**

The PROMIS scales are administered at the University of Rochester during routine clinical care. Patients with current procedural codes consistent with ankle arthrodesis and data of at least 4 months or more were included (n=68). A minimum 4 month follow up was determined by examining recovery curves for PF and PI for ALL available data(>600 points). This resulted in an average follow up of 362 days (range 123–1123 days). The proportion of patients 1 standard deviation (SD) worse than normal, between 1 SD worse than normal and 1 SD above normal was calculated for pre-operative and post-operative follow-up points. Chi-square analysis was used to compare the proportions between time points. The proportion of patients improving by at least a 0.5 SD in PF, PI, or either PF or PI was also reported.

**Results**

Preoperatively patients 1 SD worse than normal totaled 24.2% for Depression, 72.1% for PF and 75.0% for PI. Post operatively patients 1SD worse than normal was significantly lower for Depression (15.2% p<0.01), PF (45.6% p<0.01) and PI (35.3% p<0.01). Except for Depression, these proportions were higher than population norms by 2.9 and 2.2, for PF and PI, respectively. A 0.5 SD improvement was achieved in PI for 58.8%, and for 52.9% in PF; And either PF or PI was improved by 0.5 SD in 67.6% of patients.

**Conclusions**

A majority of patients improve in PF and PI a 0.5 SD and achieve final scores within 1 SD of population norms. These data are likely helpful to patients/providers pre-operatively and post-operatively as they make clinical decisions associated with whether to have ankle arthrodesis surgery and determine whether patients are meeting expected recovery benchmarks.

**Table 1 (abstract P6).**
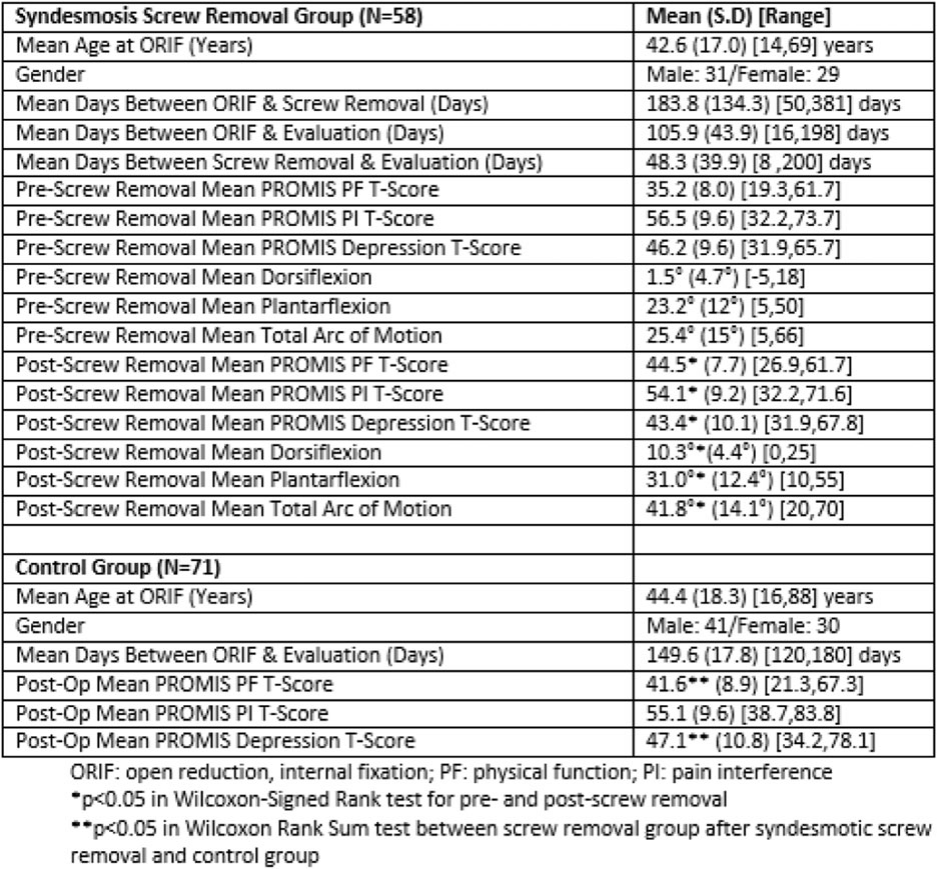
Demographic and PROMIS Data for Patients Undergoing Ankle Open Reduction and Internal Fixation (ORIF) with Syndesmotic Fixation

## P7. Are global pain interference, physical function and depression important problems in patients with diabetic foot ulcers?

### Olivia Waldman^1^, Jeff R. Houck^2^, Stephanie Hao^1^, Nicolette Lee^3^, Judith Baumhauer^1^, Irvin Oh^1^

#### ^1^Department of Orthopaedics and Rehabilitation, University of Rochester, Rochester, NY, USA; ^2^ Department of Physical Therapy, George Fox University, Newberg, OR, USA; ^3^ Sydney Kimmel Medical College at Thomas Jefferson University, Philadelphia, PA, USA

##### **Correspondence:** Judith Baumhauer (judy_baumhauer@urmc.rochester.edu)

**Background**

Diabetic foot ulcer (DFU) patients present with varying reports of pain. Many patients describe numbness, yet persistent pain secondary to advanced peripheral neuropathy. Painful diabetic peripheral neuropathy (PDPN) is one of the most common complications of diabetes, but underdiagnosed and remains poorly understood.

**Objective**

The objective was to investigate changes in DFU patient’s pain perception by analyzing PROMIS pain interference (PI), physical function(PF), and depression(D) scores before and after foot ulcer treatment. The hypotheses were that due to PDPN, a majority of DFU patients will have high baseline PROMIS PI scores, that remain unchanged by surgical intervention. Moreover, those with high PROMIS PI scores are likely to report low PF and increased depression.

**Methods**

Prospectively collected PROMIS physical function (PF), pain interference (PI), and depression scores were obtained for patients who underwent a procedural intervention for an infected DFU between February 2015 and November 2018 (n=240). Patients with at least 3 consecutive visits, a minimum post-procedural follow-up of 3 months and completion of PROMIS surveys for each visit were included in this study (n=92). Demographics, BMI, medical comorbidities, Hemoglobin A1C, procedures performed, and wound healing status data were collected. Chi-Square test, Spearman’s rank correlation coefficient, and minimum clinically important differences (MCID) were calculated.

**Results**

Eighty percent of participants were males (n=74) with an average age of 60.5 (range, 33 to 96) and BMI of 34.1 (range, 22.0 to 57.5). The average follow-up period was 4.7 (range, 3 to 12) months. Preoperatively a majority (57.6% and 76.5%, respectively) of patients reported PI and PF at least one standard deviations (SD) worse than the US average. Average change on all PROMIS scales was less than 1.7 t-score points. Patients with depression were more likely to have lower PF (*p*=0.007) and higher PI (*p*=0.001).

**Conclusions**

Despite PDPN causing numbness, a majority (57.6%) of patients with PDPN reported PI greater than 1 SD worse than the US population normal. The effects of PDPN and DFU on function are significant, resulting in PF levels that imply difficulty with daily activities. Depression is an important symptom for clinicians to focus on as patient function declines and pain increase.

## P8. Derivation via scoring service of PROMIS® fatigue scores based on FACIT with Sarilumab in rheumatoid arthritis

### Clifton O. Bingham III^1^, Susan Boklage^2^, Toshio Kimura^2^, David Cella^3^

#### ^1^Johns Hopkins University, Baltimore, MD, USA; ^2^Regeneron, Tarrytown, New York, NY, USA; ^3^Northwestern University, Chicago, IL, USA

##### **Correspondence:** Clifton O. Bingham III (cbingha2@jhmi.edu)

**Background**

There is growing interest in the Patient Reported Outcome Measurement Information System® (PROMIS®) for the assessment of fatigue, a prevalent symptom of rheumatoid arthritis (RA). This study derived PROMIS-Fatigue 13- and 10-item scores from individual patient-level FACIT-Fatigue scores, via the publicly available scoring service (http://www.healthmeasures.net), to assess treatment effect from 3 global Phase 3 trials with sarilumab. Sarilumab, a fully human monoclonal antibody directed against IL-6Rα, is for treatment of moderately-to-severely active RA. Sarilumab 150mg or 200mg subcutaneous (SC) every 2 weeks (Q2W) vs placebo was assessed in tumor-necrosis-factor-inhibitor-irresponsive patients (24-week TARGET; n=546), methotrexate-irresponsive patients (52-week MOBILITY; n=1197) and as monotherapy with sarilumab 200mg vs adalimumab 40mg SC Q2W (24-week MONARCH; n=369).

**Methods**

For each trial and treatment arm, least-square mean (LSM) Week 24 change-from-baseline (CFB) FACIT-Fatigue, PROMIS-Fatigue 13- and 10-item scores were obtained for sarilumab 200mg, placebo and adalimumab 40mg, globally and by study region: South America (S-America), Western Europe (W-Europe), Eastern Europe (E-Europe), Asia, Australasia and Africa.

**Results**

Baseline PROMIS-Fatigue 13- and 10-item scores were similar per trial. Respective FACIT-Fatigue//PROMIS-Fatigue scores, differing regionally, ranged between 20.84–24.4//58.77–62.85, 24.92–30.64//55.27–59.50 and 22.35–25.50//58.48–61.58 across the regions in TARGET, MOBILITY and MONARCH, respectively, indicating high levels of baseline fatigue. FACIT-Fatigue//PROMIS-Fatigue 13 item LSM-CFB scores: were 6.82//–4.69 and 5.80//–4.37 for placebo; 10.06//–6.63 and 9.15//–6.65 for sarilumab (TARGET and MOBILITY, respectively); and 8.41//–5.56 for adalimumab and 10.18//–6.71 for sarilumab (MONARCH). Regionally, FACIT-Fatigue//PROMIS-Fatigue 13 item LSM-CFB scores ranged from 2.34//–1.93(E-Europe) to 11.61/–8.03 (Asia) for placebo, 6.78//–3.67(N-America) to 13.16(S-America)//–9.83(Asia) for sarilumab in TARGET; 5.03(Asia)//–3.03(W-Europe) to 7.23(Africa)//–5.17(S-America) for placebo, 7.34(N-America)//–4.87(Australasia) to 11.54(Australasia)//–7.56(W-Europe) for sarilumab in MOBILITY; 4.93(N-America)//–3.75(N-America) to 13.33//–8.69(Africa) for adalimumab, and 4.33(Africa)//–2.43(Africa) to 11.75(W-Europe)//–8.96(N-America) for sarilumab in MONARCH. Treatment-by-region interaction tests, in which no nominal P-values were less than 0.05, suggested that there were no treatment differences across the regions, nor were there variations in baseline scores that could explain the CFB differences.

**Conclusions**

Although baseline mean values for and PROMIS-Fatigue scores differed across regions, the treatment effect across regions was comparable.

**Funding**

The study was funded by Sanofi and Regeneron Pharmaceuticals, Inc.

**Conflict of interest**

S. Boklage and T. Kimura are employees and shareholders in Regeneron Pharmaceuticals, Inc. D. Cella is a consultant to Sanofi and Regeneron Pharmaceuticals. C. Bingham is a consultant to Sanofi and Regeneron.

Acknowledgments: Medical writing support was provided by Gauri Saal, MA Economics, Prime, Knutsford, UK and was funded by Sanofi and Regeneron Pharmaceuticals, Inc.

## P9. Anxiety and depression symptoms increase office telephone communication for patients with rheumatoid arthritis

### Dana DiRenzo, Michael Wu, Thomas Grader-Beck, Clifton O. Bingham III

#### Johns Hopkins University, Baltimore, MD, USA

##### **Correspondence:** Clifton O. Bingham III (cbingha2@jhmi.edu)

**Background**

We hypothesized mental health disturbances in patients with Rheumatoid Arthritis (RA) would significantly impact office communication volume. We evaluated telephone call volume in RA patients who completed the Patient Reported Outcome Measurement Information System (PROMIS) Global Profile-29 during routine clinical care.

**Methods**

A convenience sample of 530 medical records of patients who received care at a university specialty care center were screened. RA patients who received care for at least 1 year duration and completed the PROMIS Global-29 profile during routine appointments between April and September 2018 were identified; telephone call encounters were retrospectively totaled for 1 year. Patients were stratified by symptoms of high and low anxiety and depression symptoms based on PROMIS anxiety and depression T-scores that were </>= 60. Telephone call volume was compared between groups using student’s t-tests and a multivariable linear regression model was constructed to evaluate the association with high anxiety or depression symptoms.

**Results**

182 RA patients met the 2010 American College of Rheumatology classification criteria and had profiles completed. Patients were mostly female (n=131, 72%) and white (n=127, 70%) with a mean age (SD) of 59 (14). The median RA disease duration (SD) was 8.5 years (5, 16) and median CDAI (IQR) was moderate at 7.5 (4, 18). High anxiety symptoms were present in 26% of RA patients (n=47), and high depression symptoms in 20% (n=36). RA patients with high vs low anxiety or depression symptoms had significantly more telephone encounters (7.3 (11.5) vs 3.0 (5.1), p=0.0179; 8 (11.8) vs 3.1 (5.6), p=0.0217). Anxiety symptoms and depression symptoms both were significantly associated with telephone communication volume in multivariable regression (β=0.209, p=0.014; β=0.180, p=0.034). However, when controlling for use of a mood stabilizer, anxiety and depression symptoms no longer impacted communication volume.

**Conclusions**

This study demonstrates the ability of PROMIS measures to identify patients with high levels of anxiety and depressive symptoms in routine care settings. Identification of anxiety and depression symptoms may prompt implementation of multi-modal strategies to address these symptoms and to provide additional information to alleviate patient concerns regarding their health.

## P10. A real-world evidence-based assessment and intra-method correlative analysis of PROMIS-29, pain interference short form 6b, fatigue short form 7a, with the clinical disease activity index and sf-36 questionnaire among a community-based rheumatoid arthritis population

### Clifton O. Bingham III^1^, Shelly Kafka^2^, Shawn Black^2^, Stephen Xu^3^ and Jeffrey R. Curtis^4^

#### ^1^Johns Hopkins University, Baltimore, MD, USA; ^2^Janssen Scientific Affairs, LLC. Horsham, PA, USA; ^3^Janssen Research & Development, LLC. Spring House, PA, USA; ^4^University of Alabama at Birmingham, Birmingham, AL, USA

##### **Correspondence:** Clifton O. Bingham III (cbingha2@jhmi.edu)

**Objective**

Use of PROs to assess health-related quality of life in clinical practice, research studies, and clinical trials in Rheumatoid Arthritis (RA) remains an ongoing area of research. The Clinical Disease Activity Index (CDAI) utilized in rheumatology practice is derived from four components including Physician Global Assessment, Patient Global Assessment, Swollen and Tender joint counts (from a total of 28 individual joints, with a maximum score of 76). SF36 is commonly used in RA trials but is not easily adaptable for practice settings to guide care. PROMIS (Patient Reported Outcomes Measurement Information System) may address this gap but has not been widely assessed in RA patients starting therapy in real world clinical practice or compared with overall or component CDAI scores. These were evaluated in the AWARE (Comparative and Pragmatic Study of Golimumab IV Versus Infliximab in Rheumatoid Arthritis) study, an ongoing Phase 4 study designed to provide a real-world assessment of intravenous Tumor Necrosis Factor inhibitor (TNFi) medications in RA pts.

**Methods**

AWARE is a prospective, noninterventional, 3-year study at 88 US sites. RA pts were enrolled when initiating TNFi treatment. All treatment decisions were made by the treating rheumatologist, with PROMIS and SF 36 assessments made at baseline, the 2^nd^, 5^th^ and 8^th^ infusion. CDAI assessments were made at baseline, 3, 6 and 12 months, and then every 6 months. Here we report baseline PROMIS-29 (7 domains and pain intensity), PROMIS Pain Interference (PI) Short Form (SF) 6b (PI6b) and PROMIS Fatigue (F) Short Form 7a (F7a), domain T-Scores, CDAI, SF36 subdomain and Component Scores (CS) in 1270 RA patients enrolled in the AWARE study. Baseline study data are reported here. Correlations between PROMIS measures and (1) CDAI component and total scores and (2) comparable SF36 component scores were calculated using Pearson Correlations. Data are mean ± standard deviation (SD).

**Results**

At baseline, the mean CDAI of all pts (n=1262) was 32.2±15.597. The % of pts with High Disease Activity (CDAI>22), Moderate Disease Activity (CDAI >10 and ≤22), Low Disease Activity (>2.8 and ≤10) and Remission (<=2.8) was 70.4, 22.8, 6.1 and 0.7, respectively. Mean PROMIS-29 T-scores (except Anxiety and Sleep Disturbance) among patients with HDA were significantly different from patients with MDA, LDA or remission. Further, mean PROMIS T-scores of PF, F, PSRA, PI, Pain Intensity, PI6b and P7a among patients with MDA, were significantly different from patients with more or less severe RA (by categorical CDAI). Among CDAI component scores, the Patient Global Assessment was consistently the most highly correlated with PROMIS T-scores among the four CDAI components (Tender Joint count, Swollen joint count, patient global assessment and physician global assessment); the most highly correlated among the PROMIS-29 domains were Pain Intensity (0.743), Pain Interference (0.645). PI6b had a Pearson correlation of 0.647 for Patient Global assessment. The mean baseline P29 Depression and Anxiety T-scores were within 0.5 SDs of respective population means. P29 scores were >0.5 SD worse than population means for Physical Function (PF, 38.1±6.84), PI (63.4±7.68), F (58.8±9.95), Sleep Disturbance (55.1±8.68); Ability to Participate in Social Roles/Activities (PSRA, 43.4±8.58). PI6b, F7a, and P29 domain T-scores were highly correlated with the comparable SF36 subdomain and component scores (r’s >0.58), excepting sleep, for which no comparable SF36 element was applicable. Examples include: PI6b (r=0.-796) and P29-PI (0.807) with SF-36-Bodily Pain; F7a (-0.765) and P29-F (-0.774) with SF36-Vitality; P29-PF with SF36-PF (0.766), Role-Physical (0.688), and Physical CS (0.731); P29 Anxiety with SF36-Mental Health (-0.715), Role- Emotional (-0.559), Mental CS (-0.695); and P29-PRSA with SF36-Social Functioning (0.705).

**Conclusions**

High correlations between individual PROMIS29 domain T-scores and SF36 component scores, and categorical CDAI, provide strong evidence of PROMIS construct validity in a real-world population of RA patients.

## P11. The EPIC PROMIS: implementing routine PROMS collection in orthopaedics

### Jeanne T. Black, Marco Castro, Mark Vrahas

#### Cedars-Sinai Medical Center, Los Angeles, CA, USA– all authors

##### **Correspondence:** Jeanne T. Black (Jeanne.Black@cshs.org); Marco Castro (Marco.Castro@cshs.org)

**Background**

Despite CMS mandates and growing recognition that PROMs are important in assessing value, few institutions have successfully implemented their routine collection. The Orthopaedic Department at Cedars-Sinai committed to collecting PROMS routinely across all subspecialties. This report describes our implementation strategy and results.

**Methods**

Key components of our strategy were 1) Obtain high level organizational support and resources for implementation; 2) Demonstrate leadership’s ongoing commitment; 3) Minimize disruption to clinical flow and providers; 4) Offer patients options for survey completion. Orthopaedic leadership garnered commitment from senior management to dedicate necessary IT department resources. The Orthopaedic Chairman made multiple presentations to clinic staff and physicians, explaining the initiative and its importance. Ongoing reinforcement is provided through continuing Chairman presentations, weekly reports to clinic managers on collection rates, monthly staff lunches when rates exceed goal, and monthly reports to physicians showing their collection rate compared to other physicians in their clinic. The orthopaedic project manager visited 3 institutions where routine PROM collection had been implemented to understand their workflows and analyzed Cedars-Sinai workflows. After reviewing several platforms, the team agreed that PROMIS computer adaptive tests (CAT) collected directly on EPIC’s platform would best satisfy our goal of minimal disruption to clinic operations while allowing the collection of a standard set of instruments relevant to all orthopaedic subspecialties (Physical Function, Pain Interference, Depression). Patients have two options to complete the PROMs, online using EPIC’s MyChart and/or at their clinic visit. Patients can complete the PROMIS CAT in Epic’s Hyperspace, using the exam room clinical workstation, locked so only the questionnaire is visible. Physicians can view patient t-scores and graphed results immediately in Epic and use a SmartLink to pull the t-scores into their progress notes.

**Results**

The original collection goal of 75% was achieved in the first 3 months, with an average of 78%. The goal was increased to 80%; for the subsequent 9 months, clinics collected PROMs for 83% of patients. Patients completed 10% of PROMS via MyChart and 90% during their clinic visits.

**Conclusions**

A multi-pronged strategy was essential to our success. Continued reinforcement is required to maintain high collection rates.

## O12. A new measure from the PROMIS adult physical function item bank: developing and validating clinician-reported inpatient physical function

### Heather E. Brown^1^, Michael A. Kallen ^2^, Joeffrey R. Hatton^1^, William A. Doyle^1^, Ryan Murphy^1^, Ryan Elliott^1^, Ann T. Tran^1^, Mark A. Gutierrez^1^, John D. Litten^1^, Richard C. Gershon, PhD^2^, Vincent X. Liu^1^

#### ^1^ The Permanente Medical Group, Kaiser Permanente; Oakland, CA, USA; ^2^ Northwestern University Department of Medical Social Sciences, Chicago, IL, USA

##### **Correspondence:** Heather E. Brown (Heather.E.Brown@kp.org)

**Background**

We aimed to develop and validate a precise, score-level targeted clinician-reported inpatient physical function (PF) measure. Item content was derived from PROMIS PF item bank content and scores reported on the PROMIS PF metric.

**Methods**

The PROMIS PF item bank was reviewed by psychometricians and clinicians to identify items measuring lower-level PF (T-scores 10-50) that, collectively, offered high score-level reliability (≥ 0.90), suggested by established PROMIS PF item performance. Selected items were edited for clinician reporting, reviewed by external clinicians, and field tested. Response data were assessed for meeting measure development standards; items were calibrated on the PROMIS PF metric via a single-group design linking study, using patient-reported responses to the PROMIS PF items identified for clinician reporting. A 5-item short form (SF) was constructed and new clinical data analyzed for validity evidence.

**Results**

Nine PROMIS PF items were candidates for clinician reporting of inpatient PF; three new items were written to extend content coverage. (Table 1.) N = 515 inpatients (55.1% female; mean age = 66.2 years) were assessed by physical therapists using the 12 inpatient PF items. Response data analyses indicated the items met expected measure development standards (e.g., eigenvalue 1 = 83.8% of variance; Cronbach’s alpha = 0.97). The 12 inpatient PF items were linked to the PROMIS PF metric, using inpatient responses to the nine original PROMIS PF items for anchoring (raw score *r* = 0.73). The 5-item SF assesses inpatient PF from T-scores 10-60 (score-level reliabilities ≥ 0.90 for T-scores 10-45). Validation study (N = 481) median SF T-scores were 35.8 (IQR 28.8-39.9) for inpatients discharged home without home health, 30.4 (IQR 26.0-34.0) for those discharged with home health, and 21.9 (IQR 17.3-26.0) for those with other discharge dispositions. The SF demonstrated very good to excellent discrimination for inpatient discharge home without home health (46.8% of inpatients; c-statistic 0.78) and home including home health (76.1% of inpatients; c-statistic 0.87), compared with other discharge dispositions.

**Conclusions**

We developed and validated a precise, score-level targeted measure for clinician reporting of inpatient PF; its 5-item SF renders this measure an effective, efficient means of assessing inpatient PF.

## P13. Validation of the Arabic version of PROMIS-10 global health assessment in a Swedish immigrant population

### Susan Ghalayini^1^, John E Chaplin^2^

#### ^1^ Institute of Medicine, Gothenburg University, Gothenburg, Sweden; ^2^ Institute of Clinical Science, Gothenburg University, Gothenburg, Sweden

##### **Correspondence:** John E Chaplin (john.chaplin@gu.se)

**Background**

Given the level of global migration, it is increasing necessary to identify valid instruments for the measurement of health in immigrant populations. The objective is to test the validity of the Arabic version of PROMIS-10 in an immigrant population in Sweden.

**Methods**

Data using the Arabic versions of the PROMIS-10, Hospital Anxiety & Depression scale (HADS) and socio-demographic background questions were collected via an internet survey tool. Adults over the age of 18 were contacted in Gothenburg City, Sweden. The data were collected via an online survey. The link to the survey was sent via social media groups to people living in Gothenburg, email and via handouts to people in shopping malls. Internal consistency of individual items with the overall score was assessed using Cronbach's alpha coefficient. Construct validity was evaluated by determining Spearman's correlation between the Arabic PROMIS-10 score and scores from the HADS Physical and Mental health. Arabic, Swedish and English versions were available on-line.

**Results**

There were 106 Arabic versions of the questionnaire completed (72% female) with a further 30 Swedish and 10 English. 125 people classified themselves as an immigrant (86%) with 79% from Arabic countries. 72% of respondents had been in Sweden for less than 5 years; 95% with high school education or higher; 37% were in full-time employment. From the cut-off score for the HADS 35% were anxious and 6% depressed. Internal consistency for Physical Health was 0.804, and Mental Health 0.824. Construct validity for Physical health - HADS Anxiety -0.423; HADS Depression -0.513; Mental health – HADS Anxiety -0.734; HADS Depression -0.670.

**Conclusions**

The PROMIS-10 Arabic version has good internal consistency in an immigrant population in Sweden. The GH-mental health scale appears to be valid against the HADS mental health score. The instrument retains the characteristics of the original English and Swedish language versions.

## O14. Checking the metric: PROMIS domain short form equivalence, scoring methods and the impact of missing data

### Robert Chapman, Benjamin D. Schalet, Kathryn Jackson

#### Northwestern University, Department of Medical Social Sciences, Chicago, IL, USA

##### **Correspondence:** Robert Chapman (robert.chapman@northwestern.edu)

**Background**

PROMIS measures can be administrated with a variety of item content and test lengths, have multiple scoring methods, but simplify to a common metric. The multiplicity of PROMIS scoring options and test lengths allows users to accommodate to the realities of clinical or population-based research, balancing measurement error and patient burden. However, it is unclear to what extent the tests and scoring methods are interchangeable. This work evaluates the equivalence of PROMIS profile domain short forms and scoring methods across research contexts and levels of missing data. We offer recommendations for managing group-level missing data.

**Methods**

Analyses were conducted in three “studies”. Study 1 used simulation datasets to examine scale-level score agreement (ICC) and error across short forms and scoring methods (IRT pattern response, look-up table). Study 2 evaluated agreement and error among short forms and scoring methods in both clinical and general population empirical datasets. Study 3 examined intra-individual missing data, data imputation methods and differences in custom short form parameters.

**Results**

In Study 1, we simulated 1,000 model scores directly from IRT parameters. Multiple short forms and scoring methods showed excellent agreement with each other (ICC2 0.95-0.99) and minimal error (2.28-5.11 RMSE T-score units), with no clear preference for pattern vs look-up table scoring. Results were similar in empirical data sets (ICC2 0.90-0.97, 0.59-5.55 RMSE). When missing item data was induced, differences between short forms and scoring methods emerged, with longer pattern scored short forms best minimizing error, but look-up table scoring with missing data and mean-item substitution showing little additional bias or disagreement. Across both scoring methods, shorter measures showed twice the rate of error of longer measures.

**Conclusions**

PROMIS profile domain short forms stay close to the true metric by producing equivalent and reliable scores across short forms, scoring methods and research contexts. Pattern response scoring is recommended as a “gold standard” scoring, due to its flexibility, marginally better score stability across short forms and relative insensitivity to missing data. However, look-up table scoring is a valid alternative, even when item-level data are missing and parameters vary across short forms.

## P15. Chinese children’s health Status in the initial three months of cancer treatment

### Lei Cheng^1^, Ying Gu^2^, Jiashu Wang^3^, Wen Zhang^1^, Yingwen Wang^2^, Changrong Yuan^1^

#### ^1^School of Nursing, Fudan University, Shanghai, China; ^2^Children’s Hospital of Fudan University, Shanghai, China; ^3^Shanghai University of Medicine&Health Sciences, Shanghai, China

##### **Correspondence:** Lei Cheng (chenglei@fudan.edu.cn)

**Background**

Children with cancer suffers from symptoms and function changes during their disease continuum. However, there were limited self-reported data about their health status in the initial three months of cancer treatment. The aim of the study was to measure patient-reported outcomes (PROs) in children with cancer in the initial three months of cancer treatment and factors that potentially were associated with their symptoms and function level.

**Methods**

Children aged 5-18 years, newly diagnosed with cancer were enrolled. The Pediatric Patient-Reported Outcome Measurement Information System (PROMIS) was used to measure anxiety, depression, fatigue, anger, pain interference, mobility, upper extremity function, and peer relationship. Test statistics and ANOVA were used to evaluate relationships between PROMIS measures and potentially influential variables.

**Results**

A total of 131 children of 5-18 years (mean age = 8.62 years; 64.90% males), completed the survey, 61.4% had leukemia/lymphoma. Most of the PRO symptom scores were positively correlated, but negatively correlated with functional scores, except for peer relationships. Male patients reported higher fatigue and lower peer relationship. Children undergoing radiotherapy reported highest fatigue. Younger children (less than 8 years old) reported higher anxiety and pain interference, but lower upper extremity function. Children who admitted to hospital more than twice reported lower peer relationships. (All *p*<0.05)

**Conclusions**

Understanding the burden of cancer treatment in the initial three months is critical to refine supportive care interventions to minimize the burden of pediatric cancer treatment. Clinicians need to be aware of the significant associations found between children’s PROs and clinical as well as demographic characteristics.

## O16. Measurement characteristics of PROMIS Computer Adaptive Testing (CAT) fatigue and ESASr tiredness in kidney transplant

### Sumaya Dano^1^, Evan Tang^1^, Gauree Chavla^1^, Niroban Jayakumar^1^, Areej Ali^1^, Susan J. Bartlett^2^, Madeline Li^3^,

Doris Howell^3^, John D. Peipert^4^, Marta Novak^5^, Istvan Mucsi^1^

#### ^1^Multi-Organ Transplant Program, University Health Network, Toronto, Canada; ^2^ Dept. of Clinical Epidemiology, Research Institute of the McGill University Health Centre, Montreal, Canada; ^3^Princess Margaret Cancer Centre, Toronto, Canada; ^4^Department of Medical Social Sciences, Feinberg School of Medicine, Northwestern University, Chicago, IL, USA; ^5^Centre for Mental Health, University Health Network, Toronto, Canada

##### **Correspondence:** Sumaya Dano (sumaya.dano@mail.utoronto.ca)

**Background**

Fatigue is a common and debilitating symptom and is currently not screened for systematically in organ transplant in part due to the lack of sensitive and feasible tools. In this study we evaluate and compare the diagnostic accuracy of the NIH Patient Reported Outcomes Measurement Information System CAT fatigue item bank (PROMIS-F CAT) and Edmonton Symptom Assessment System fatigue (ESASr-F) scores in kidney transplant recipients (KTR).

**Methods**

A cross-sectional, convenience sample of adult outpatient KTR completed PROMISF CAT, the ESASr and the Functional Assessment of Chronic Illness Therapy-Fatigue (FACITF) scale on an electronic data capture system. Spearman’s rho was used to report correlations, and receiver operating characteristic (ROC) analysis was used to assess discrimination of

ESASr-F and PROMIS-F CAT, using the FACIT-F scale score ≤30 as a diagnostic criterion for moderate/severe fatigue. Cut-off scores were identified using the Youden index. In a different set of analyses, the PROsetta Stone crosswalk was used to determine the PROMIS-F CAT cut off score that corresponds to the FACIT-F cut-off.

**Results**

Among 65 KTR patients (mean [SD] age 54[13] years, 38 males [58%]), 11% had moderate to severe fatigue based on the FACIT-F score. Correlations between FACIT-F and PROMIS-F CAT (rho=-0.68; p<0.01) and ESASr-F (rho=-0.68; p<0.01) were strong. The PROMIS-F CAT had numerically higher discrimination compared to ESASr-F, however the difference was not statistically significant (area under the curve: PROMIS-F CAT =0.93, CI:0.83-1; ESASr-F =0.86, CI:0.62-1; p=0.66). The PROMIS-F CAT cut-off score for significant fatigue identified by the Youden index was 57 (Sensitivity=86%, Specificity=88%, Positive Predictive Value (PPV)=0.46, Negative Predictive Value (NPV)= 0.98); the cut-off score for ESASr-F was 6 (Sensitivity=86% Specificity=93%, PPV=0.6, NPV=0.98). The PROMIS-F CAT cut-off score based on the ROC curve analysis was similar (57.8) to the cut-off determined using the PROsetta Stone crosswalk file.

**Conclusions**

In this analysis we found that the PROMIS-F CAT and ESASr-F had similar discrimination for significant fatigue. We also identified clinically useful cut-offs for significant fatigue for PROMIS-F CAT and ESASr-F. The results suggest that both PROMIS-F CAT and ESASr-F may be potentially feasible as a screening tools for fatigue among KTRs.

## P17. Do utility elicitations preserve the psychometric benefits of PROMIS® item banks?

### Barry Dewitt^1^, Janel Hanmer^2^

#### ^1^Department of Engineering & Public Policy, Carnegie Mellon University, Pittsburgh, PA, USA; ^2^Division of General Internal Medicine, University of Pittsburgh, Pittsburgh, PA, USA

##### **Correspondence:** Barry Dewitt (barrydewitt@cmu.edu)

**Background**

Health utility scores map health states to a unidimensional construct, utility, that compactly represents preferences for those health states. For utility elicitation tasks that are used to create the score mappings, health states are described using multi-dimensional qualitative descriptions, where each state is described using levels of health domains (e.g.,

physical function, depression, fatigue). The PROMIS®-Preference (PROPr) score is the first health utility score mapped from item banks. Item response theory guarantees the commensurability of different items from the same item bank. However, no one knows whether, in practice, that commensurability is preserved in utility elicitation tasks.

**Methods**

PROPr is constructed from seven PROMIS domains. In utility elicitation tasks, each domain is represented with two items from its item bank. We compare utility elicitations that produced the PROPr score (n=983) with a second online panel (n=630) that undertook the same preference elicitation task, but with different items from three of the item banks: Cognitive Function-Abilities (*cognition*), Depression (*depression*), and Pain Interference (*pain*). We use beta regression to determine the size of the item-choice effect on single-attribute utility functions.

**Results**

The choice of items does not affect the utility function for the depression domain. For the cognition domain, it affects the curvature of the utility function but not its estimate of the population mean value of cognition (coefficient = 0.28, logit scale, p<0.01). For the pain domain, it affects the mean utility estimate for the population mean value of pain but not the curvature of the utility function (coefficient = -0.39, p<0.01).

**Conclusions**

Two of the three domains showed little item-choice effect on the most important part of the utility function, its curvature. Thus, to some extent, utility elicitations preserve the commensurability of items within an item bank. Future research should quantify the degree to which the standard gamble utility elicitation, its particular implementation in this study, and participant characteristics affect these results.

## O18. Preoperative PROMIS 10 physical function scores help predict opioid dependence after lumbar fusion surgery

### Terence P Doorly^1^, Rachel C Sisodia^2^

#### ^1^NSPG Spine Program, Peabody, MA, USA; ^**2**^Massachusetts General Hospital & Partners HealthCare System, Boston, MA, USA

##### **Correspondence:** Terence P Doorly (tdoorly@partners.org)

**Objective**

Opioid naïve patients undergoing lumbar fusion surgery are seldom informed that opioid dependence is a potential postoperative outcome. We sought to determine the incidence of postoperative opioid dependence in this population, and to evaluate preoperative PROMIS 10 physical function scores as a tool to help predict those patients at higher risk of opioid dependence.

**Methods**

Patients undergoing spinal fusion surgery are routinely administered the following surveys - PROMIS 10, PROMIS physical function and PROMIS pain interference, preoperatively and at 30, 90, 180 and 365 days postoperatively. During the period January 1, 2014, to October 1, 2018, data was collected on 95% of eligible patients. For this study, patients were selected for analysis if at the time of surgery they were opioid naïve and had not filled a narcotic prescription within the previous year, as validated by state and federal prescriber monitoring databases. Post-operative opioid use was validated using the same databases. Patients were defined as opioid dependent if they were still filling narcotic prescriptions 6 months after surgery. Appropriate statistical analysis was performed.

**Results**

Paired pre and postoperative data were available on 802 patients. 734 patients (92%) were opioid naïve prior to surgery. Of these, 4 patients (0.5%) scored above average on pre-operative PROMIS 10 physical function scores, 197 patients (26.8%) scored in the average range and 533 (72.6%) scored below average. 68 patients (9%), opioid naïve prior to surgery, were still filling narcotic prescriptions more than 6 months after surgery. 10.5% of patients with below average and 6% of patients with average pre-operative PROMIS 10 physical function score became opioid dependent. No patient with above average PROMIS 10 physical function scores became opioid dependent.

**Conclusions**

Currently, 9% of our opioid naïve patients undergoing lumbar fusion surgery become opioid dependent postoperatively. PROMIS 10 physical function scores help predict the risk of this complication, and when used in conjunction with other validated tools, enhance shared decision making prior to surgery, and direct judicious prescribing of narcotics postoperatively.

## P19. Use of Caregiver-Selected PROMIS measures in the evaluation a pragmatic clinical trial for children with medical complexity

### Nora Fayed^1^, Julia Orkin^2^, Nathalie Major-Cook^3^, Audrey Lim^4^, Eyal Cohen^2^

#### ^1^Queen’s University, Kingston, Ontario, Canada; ^2^The Hospital for Sick Children, Toronto, Ontario, Canada; ^3^Children’s Hospital of Eastern Ontario, Ottawa, Ontario, Canada; ^4^McMaster Children’s Hospital, Hamilton, Ontario, Canada

##### **Correspondence:** Nora Fayed (nf31@queensu.ca)

**Background**

Children with Medical Complexity (CMC) have medical fragility, chronic health conditions, and elevated healthcare service use. CMC account for as little as 0.5 % of all children in Canada (~400,000 in the US), yet they use about one-third of all child health resources. A pragmatic clinical trial for CMC, is evaluating the roll-out of a coordinated care intervention to meet CMC needs. Caregivers and expert clinician providers set out to develop an evaluation framework to guide the selection of primary and secondary trial endpoints.

**Methods**

Literature review of outcomes, caregiver and clinician engagement survey, and consensus conference methods were used to determine the core set of outcomes requiring measurement in the trial. Outcomes were assessed by caregivers and clinicians based on their importance and amenability for change due to intervention.

**Results**

The primary endpoint, ‘experience of coordinated care’, was rated by caregivers and clinicians as the most important and amenable for change. Caregiver sleep, energy and fatigue and mood were prioritized as secondary endpoints, and PROMIS tools were endorsed for use in the trial to measure them.

**Conclusions**

PROMIS tools that measure sleep, energy and fatigue and mood were endorsed as important and amenable to change in the context of clinical trials for caregivers of medically complex children. Trial results as evaluated by PROMIS tools will be available September 2020.

## P20. Reducing respondent burden for spine patients – developing a prediction model for ODI and COMI based on PROMIS 29

### Claudia Hartmann, Felix Fischer

#### ^1^Center of Internal Medicine and Dermatology, Department of Psychosomatic Medicine, Charité –Universitaetsmedizin Berlin, corporate member of Freie Universität Berlin, Humboldt-Universität zu, Berlin and Berlin Institute of Health, Berlin, Germany

##### **Correspondence:** Claudia Hartmann (Claudia.Hartmann@charite.de)

**Objective**

The Charité Spine Center collects several PROMs in order to meet requirements such as the Registry of the German Spine Society and the ICHOM Lower Back Pain Set. Therefore, the following quality of life measures are currently collected: ODI, EQ-5D-5L, PROMIS Profile 29 and COMI (Back and Neck). In total 65 questions are asked, 20 of them targeted to measure pain and pain intensity. In order to reduce respondent burden, we investigated whether scale

scores of the ODI and/or COMI Back could be predicted from PROMIS Profile 29.

**Methods**

We used PROMs from 350 patients undergoing treatment (surgery or none invasive) at our Spine Center care units. Data was randomly split in calibration (n = 250) and validation (n = 100) sample. First, we analyzed between-scale correlations for all of the questionnaires. We then predicted the ODI and COMI Back score by different sets of predictor variables in a

multivariate regression: all PROMIS Domain Scores, Top3 and Top2 correlated scores, and a single regression with highest correlated score. We then tested each of the three prediction models on individual and sample level in the validation sample.

**Results**

The highest correlations (r = -0.87) was found between ODI Score and PROMIS Physical function and between COMI Back Score and PROMIS Pain Interference (r = 0.70). The regression models for the ODI showed that for an individual prediction the root-mean-square deviation varies from RMSE single regression = 12.92 and evolves by adding variables to 9.69 (Top3). For COMI Back the RMSE for single regression is 1.50 and evolves to 1.477 for multi regression (Top3).

**Conclusions**

Prediction of ODI scores on a sample level using multivariate regression with 3 variables was feasible, but predictions of individual scores came with substantial error. Inclusion of predictors moderately associated with the ODI improved prediction. Prediction of COMI Back score on a sample level and for individual scores using multivariate regression with the TOP 3 correlated variables was feasible. Inclusion of predictors moderately associated with the COMI-back score moderately improved prediction.

## O21. Measuring PROMs using Computer Adaptive Testing (CAT) after an operative intervention of an extremity fracture

### Roos J.M. Havermans^1,2^, Koen W.W. Lansink^2^, Taco Gosens^3^ Mariska A.C. de Jongh^1^

#### ^1^Brabant Trauma Registry, Network Emergency Care Brabant’, Tilburg, the Netherlands; ^2^Department of Surgery, ETZ Hospital (Elisabeth-TweeSteden Ziekenhuis), Tilburg, the Netherlands; ^3^Department of Orthopaedics, ETZ Hospital (Elisabeth-TweeSteden Ziekenhuis, Tilburg, the Netherlands

##### **Correspondence:** Roos J.M. Havermans (r.havermans@etz.nl)

**Background**

Patient Reported Outcome Measurement Information System (PROMIS) is a valid measurement system and can contribute to the evaluation of health care and health related quality of life. It was designed to enhance communication between clinicians and patients. The use of Computer Adaptive Testing (CAT) based on Item Response Theory (IRT) required significantly fewer questions and less time to complete. The aim of the study is to examine the correlation between the generic PROMIS CAT questionnaires and injury specific questionnaires after an operative intervention of an upper or a lower extremity fracture.

**Methods**

A prospective cohort study was conducted. All trauma patients who underwent an operative intervention of an upper or lower extremity fracture between April and December 2018 and responded to the CAT questionnaires were included. The injury specific questionnaires are the Quick Disabilities of the Arm, Shoulder and Hand (DASH) and the Lower Extremity Functional Scale (LEFS) for the upper and lower extremity fractures respectively. The correlation between the injury specific questionnaires and the CAT questionnaires (PROMIS Physical Function (PF), PROMIS Social Function (SF) and PROMIS Pain Interference (pain)) were calculated with the Pearson’s correlation (r). Response burden and floor/ceiling effects were calculated for each questionnaire.

**Results**

A total of 420 measurements were registered, 254 in patients with an upper extremity fracture and 166 in patients with a lower extremity fracture. The Pearson’s correlation was strong between the Quick DASH and PROMIS PF (r=-0.711; p<0.001) and the LEFS and PROMIS PF (r=0.767; p<0.001). The best correlation was found between the Quick DASH and the combination of PROMIS PF and PROMIS SF (r=-0.752; p<0.001) and the LEFS and the combination of the PROMIS PF and PROMIS SF (r=0.777; p<0.001). There were no significant floor or ceiling effects and the CAT questionnaires showed a lower response burden.

**Conclusions**

In conclusion, the generic CAT questionnaires are reliable during rehabilitation after an operative intervention when compared to the Quick DASH and LEFS with a strong correlation and a lower response burden. The PROMIS physical function should be combined with the PROMIS social function.

## O22. Identifying responders to treatment

### Ron D. Hays^1^, Karen L. Spritzer^1^, Steven P. Reise^2^

#### ^1^Department of Medicine, University of California, Los Angeles, CA, USA; ^2^Department of Psychology, University of California, Los Angeles, CA, USA

##### Correspondence: Ron D. Hays (drhays@ucla.edu)

**Background**

It is important to provide guidance on options for evaluating individual change because there is confusion in the literature. For example, the U.S. Food and Drug Administration guidance document recommended identifying responders using empirical evidence from anchor-based methods. But using group-level change to identify responders leads to misclassification of patients as responders when they have not actually changed. In comparison to group change, much larger change is needed for statistically significant change in an individual’s score, because of the much larger standard errors for estimates of individual change.

**Materials and Methods**

We use two waves of data collected 3-months apart in a longitudinal observational study of 1834 chronic low back pain or neck pain patients. Average age was 49 and 74% female. We categorize people into three change groups (*got worse, stayed the same, got better*) using the reliable change index based on classical test theory (CTT) and item response theory (IRT) estimated standard errors for the 4-item PROMIS®-29 physical functioning scale.

The study was approved by the RAND Corporation Human Subjects Protection Committee (#2013-0763) and was registered as an observational study on ClinicalTrials.gov (ID:

NCT03162952).

**Results**

Seventy-eight percent *stayed the same* according to CTT estimates versus 91% based on IRT. Of the 1425 that were classified as the *same* according to CTT, 99% were also classified as the *same* by IRT. However, only 27% of the 173 people that were *worse* according to CTT were classified as such by IRT. Similarly, only 38% of the 236 people classified as *better* by CTT were also deemed *better* by IRT. The Spearman rank—order correlation between CTT and IRT categories of change was 0.54 (p = 0.0228) and Cramer's V was 0.50 (p <.0001). People who changed by a substantial amount (12-13 T-score points on average) were consistently denoted as changing by CTT and IRT.

**Conclusions**

Because CTT often classified people as changing when IRT indicated no change, the approach used has noteworthy implications for who ends up being classified as changed. Either approach is better than using the minimally important difference as the threshold, but IRT is preferred because it allows the standard error to vary across individuals.

**Acknowledgements**

This study was supported by a NIH National Center for Complementary and Integrative Health Grant No. 1U19AT007912-01.

## P23. Clinical utilization of patient reported outcomes in the upper extremity orthopaedic population: comparing the PROMIS upper extremity bank v2.0 and the QuickDASH measures

### Edward Heinle IV, Michael Suk, Joel Klena, L. Christopher Grandizio

#### Geisinger Medical Center, Danville, PA, USA

##### **Correspondence:** Edward Heinle IV (ewheinle1@geisinger.edu)

**Background/Objective**

Recent advancements in Computer Adapted Testing (CAT) technology has had a prominent effect in the realm of Patient Reported Outcome Measures (PROMs). It has shown to reduce time to completion and question burden while maintaining reliability. PROMIS Upper-Extremity bank v2.0 (UE) CAT and QuickDASH (QDASH) are PROMs that are intended to assess the physical functions in patients with upper extremity health conditions. The two scores have both been shown to be responsive over time as well as to be correlated with each other [Overbeek, 2015]. Moreover, a cross-walk table has been developed which maps the QDASH raw score onto the PROMIS metric. However, the QDASH has a usage history of a dozen years, and its progenitor, the full form DASH, has been used extensively for over 20 years. The purpose of this work is to provide a nuanced understanding of how to preserve this historical legacy as the UE CAT becomes increasingly popular. We have placed an emphasis on the Minimal Clinically Important Difference (MCID) as a clinical anchor for relating the two scores, as well as more in depth psychometric considerations.

**Methods**

A cohort of 2,822 patients who had undergone outpatient upper extremity orthopaedic surgery completed baseline and post-operative sessions for the QuickDASH survey alongside the PROMIS Upper Extremity v2.0 CAT and VAS Pain measure.

We conducted a retrospective review and analysis of the raw scores and patient demographics, as well as the completion time and number of questions answered per session.

**Results**

The QuickDASH took 0:52.05 on average, compared to the PROMIS UE v2.0’s 1:48.50. Despite the additional time, the PROMIS Upper Extremity demonstrated a higher consistency, with an average pre-operative score of 34.68 (SD 9.39) compared to the QDASH’s average of 48.54 (SD 23.01).

**Conclusion**

The PROMIS UE v2.0 CAT item bank demonstrates high reliability and internal consistency relative to the QuickDASH outcome measure. The difference in statistical reliability outweighs the greater completion time for the PROMIS UE CAT and stresses the importance of increased development in Computer Adapted Testing technology.

## O24. What PROMIS T-score thresholds discriminate when a patient reaches acceptable symptom state in primary care?

### Ryan Jacobson, Daniel Kang, Jeff Houck

#### George Fox University, Newberg, OR, USA

##### **Correspondence:** Ryan Jacobson (rjacobson@georgefox.edu)

**Background**

Clinical interpretation of PROMIS T-scores continues to be a challenge, limiting clinical application. Studies thresholding to patient acceptable symptom state (PASS)—a validated question for judging whether a patient believes their current health state is satisfactory—show thresholds at ½ to 1 standard deviation (SD) worse than the US average for certain scales (Physical Function (PF), Self-Efficacy for Symptom Management (SE), Pain Interference (PI)). The objective of this analysis was to establish PASS thresholds for PROMIS PF, SE, PI, Fatigue, and Depression, and to determine if ½ or 1 SD worse than the US average sufficiently discriminate PASS in primary care patients at intake, 3-14 days, and 45-60 days follow-up.

**Methods**

A broad spectrum of patients ages 20-97 years (mean=66.9±17.0; 52.7% female; diagnosis 20.9% endocrine, 18.2% circulatory, 17.9% musculoskeletal) attending primary care were administered 5 PROMIS scales and PASS at intake (n=368), and via phone at 3-14 days (n=235) and 45-60 days (n=234). Receiver-operator curves analysis was used to assess the optimal thresholds for determining PASS status. Area under the curve (AUC) and accuracy were calculated for each scale at each time point. To improve clinical interpretation, accuracy was also assessed for thresholds rounded to ½ or 1 SD worse than the US average and compared to optimal.

**Results**

At intake, AUC values were 0.76-0.79, except Depression which was 0.71. At 3-14 days AUC values were 0.81-0.84, and at 45-60 days 0.82-0.85, except for Depression which never exceeded 0.72. At intake, accuracy of “optimal” T-score thresholds to discriminate PASS ranged 71.2%-73.7%, except for Depression at 66.3%. At 3-14 and 45-60 days, accuracy increased 3.0-5.2% for all scales. Rounding to thresholds ½ or 1 SD worse than average lead to decrements in accuracy of ≤3.6%.

**Conclusions**

Clinicians should consider PROMIS T-score thresholds at ½ or 1 SD worse than the US average to discriminate PASS with good accuracy (>70%) at intake and follow-up, except for Depression. Accuracy marginally improves after intake up to 5.2%. Knowing these PASS thresholds enhances providers’ ability to use PROMIS scales for patient engagement and prioritizing patient symptoms across a broad spectrum of primary care patients.

## P25. Are PROMIS assessments important for determining patient acceptable symptom state in primary care?

### Ryan Jacobson, Daniel Kang, Tyler Cuddeford, Jeff Houck

#### George Fox University, Newberg, OR, USA

##### **Correspondence:** Ryan Jacobson (rjacobson@georgefox.edu)

**Background**

Assessments in primary care need to inform provider decisions or directly impact care. Complex symptom profiles, as provided by PROMIS scales, may be “nice to know” rather than actionable for providers. A limited set of PROMIS scales demonstrated utility in identifying a patient’s satisfaction with their overall symptom state (i.e. PASS question) in orthopedic patients.

**Objective**

The objective of this analysis was to establish whether a more comprehensive set of PROMIS scales across a wider spectrum of patients was able to predict PASS during a primary care encounter.

**Methods**

A broad spectrum of patients ages 20-97 years (mean=66.9±17.0; 52.7% female; diagnosis 20.9% endocrine, 18.2% circulatory, 17.9% musculoskeletal) attending primary care were administered 5 PROMIS computer adaptive scales and PASS at intake (n=368) and 45-60 days (n=234). Average number of comorbidities were 5.5±2.7. A total of 58.4% of patients were PASS yes. Univariate correlations were all significant among scales suggesting possible redundancy. Backward logistic regression was used to determine which scales best modeled PASS. Model fit was evaluated using the Hosmer-Lemeshow Test. The same analysis was run on the 45-60 day data to determine the repeatability of the analysis.

**Results**

The PROMIS SE(p<0.01), Fatigue(p=0.01) and PI(p<0.01) scales were retained in the final model. The accuracy of predicting PASS improved from 58.4% prior to applying the model to 76.4% after applying the model. Hosmer-Lemeshow test indicated adequate fit (p=0.46). The same PROMIS scales were retained for the 45-60 day data and model fit was adequate (p=0.79). The prevalence of PASS prior to applying the regression model for the 45-60 day sample was 60.2% and after was 80.5%.

**Conclusions**

PROMIS assessments across a spectrum of diagnoses are effective at assisting providers in understanding why patients’ PASS state may be negative or positive. This knowledge may prove critical in assisting providers to dissect complex symptom sets. Because PROMIS scales target key symptoms that are linked to a patient’s acceptable symptom state, specific actionable treatments addressing symptoms are needed.

## P26. Parent-child agreement on PROMIS asthma impact

### Aaron J. Kaat^1^, Raj Kumar^2^, Jin-Shei Lai^1^

#### ^1^Department of Medical Social Science, Northwestern University, Chicago, IL, USA; ^2^Allergy and Immunology, Lurie Children’s Hospital, Chicago, IL, USA

##### **Correspondence:** Aaron J. Kaat (aaron.kaat@northwestern.edu )

**Background**

The PROMIS Asthma Impact Scale (PAIS) 8-item short form has a parent-report version for ages 5-18 years, and a self-report version for ages 8-18 years. It is one of the few scales within PROMIS that is not centered on a general-population sample, but rather on a clinical sample of children with asthma. While the psychometric properties of the PAIS have been mostly well-described, much less is known about inter-rater reliability. This study aims to address this gap. It has been well-documented that parent-child agreement was poor on behavioral outcomes while moderate on physical health outcomes, and as such, we hypothesize poor-to-moderate agreement herein.

**Methods**

Children and their parents from three asthma clinical trials independently completed the PAIS. For dyads with more than one PAIS completion, one testing occasion was chosen for analyses. The reliability was calculated independently within each trial and then aggregated across trials to ensure that eligibility differences between studies did not modify reliability coefficients. Inter-rater reliability was evaluated using Krippendorff’s alpha with interval-scaled measurement (i.e., PROMIS T-scores), and with graphical analyses using Bland-Altman plots.

**Results**

There were 250 dyads across the three samples. In two samples, children rated their asthma impact more severe than their parents by 3 or 3.5 T-score points, but in the third sample parents rated asthma impact one T-score point worse, with a weighted average difference of higher self-report by one T-score point. However, there was wide variability in these differences (weighted SD of differences = 10.1). Agreement was modest (Krippendorff’s alpha = 0.43). Graphical analyses suggest that floor effects were common for both raters, even though this was a clinical sample. Floor effects occurred on 38% of parent-report data and 22% of self-report, with 16% of dyads being at the floor for both raters.

**Conclusions**

Parent-child interrater reliability on the PAIS is similar to other pediatric conditions. There is modest agreement between raters, and wide variability in the score differences. Floor effects were problematic and may have affected these reliability estimates. For these reasons, information from both informants should be gathered, when possible, to fully understand asthma impact among children and adolescents.

## P27. Further evidence on the validity of the multiple sclerosis-specific PROMIS Fatigue short form in the UK MS register population

### Paul Kamudoni^1^, Jeffrey Johns^2^, Sam Salek ^2, 3^, Dagmar Amtmann^4^,Karon Cook^5^, Jana Raab^1^, Ying Sun^1^, Oliver Guenther^1^, Rod Middleton^6^, Christian Henke^1^

#### ^1^Global Evidence & Value Development – R&D, Merck Healthcare KGaA, Darmstadt, Germany; ^2^School of Life and Medical Sciences, University of Hertfordshire, Hatfield, UK; ^3^Institute of Medicines Development, Cardiff, UK; ^4^Department of Rehabilitation Medicine, University of Washington, Seattle, WA, USA; ^5^Feinberg School of Medicine, Northwestern University, Chicago, IL USA; ^6^UK MS Register, Swansea Medical School, Swansea, UK

##### **Correspondence:** Paul Kamudoni (paul.kamudoni@merckgroup.com)

**Background**

The content validity and measurement properties of the PROMIS Fatigue short form are well established, based on several studies conducted in the USA.

**Objective**

The aim of this study was to generate further evidence on the measurement properties of the PROMIS Fatigue short form, including test-retest reliability and responsiveness, based on the UK MS Register population.

**Methods**

A 52-week prospective longitudinal study including patients with MS, with EDSS score < 7, is being carried out in the UK MS Register population. Participants are being assessed on the PROMIS Fatigue short form and other PROs at baseline, week 1, week 25 and week 52. Reliability was tested based on Internal consistency (Cronbach’s alpha), at baseline, and test-retest (ICC) reliability from baseline to week 1. Construct validity was evaluated based on convergent and known-groups validity analyses, based on a priori hypotheses, at baseline. Responsiveness was assessed based on score change from baseline to week 24, and week 52, across subgroups experiencing different levels of change based multiple anchors.

**Results**

Study participants (n = 384) had a mean age of 49.9 (SD =9.7; range = 22 to 65) years and 76.3 % were female. The mean (SD) T-score was 58.9 (SD = 9.41). The PROMIS Fatigue short form exhibited high Internal consistency (Cronbach’s alpha = 0.96) and good test-retest reliability in a subsample with a stable status (ICC = 0.9; n = 135). Convergence validity was demonstrated by moderate to strong correlations with related PRO measures (r = ±0.53 to 0.78). The short form was able to differentiate between groups of patients according to their global fatigue levels and on other criteria.

**Conclusion**

These results add to the cumulating evidence supporting the suitability and appropriateness of PROMIS Fatigue short form as a reliable and valid measure of fatigue in patients with relapsing and progressive forms of MS. The short form offers an opportunity to improve and standardize measurement of fatigue in patients with mild-moderate disability, in clinical practice as well as clinical research settings.

## O28. Evaluating the dimensional structure of the new multiple sclerosis PROMIS physical function short form.

### Paul Kamudoni^1^, Jeffrey Johns^2^, Sam Salek ^2, 3^, Dagmar Amtmann^4^, Karon Cook^5^, Jana Raab^1^, Ying Sun^1^, Oliver Guenther^1^, Rod Middleton^6^, Christian Henke^1^

#### ^1^Global Evidence & Value Development – R&D, Merck Healthcare KGaA, Darmstadt, Germany; ^2^School of Life and Medical Sciences, University of Hertfordshire, Hatfield, UK; ^3^Institute of Medicines Development, Cardiff, UK; ^4^Department of Rehabilitation Medicine, University of Washington, Seattle, WA, USA; ^5^Feinberg School of Medicine, Northwestern University, Chicago, IL, USA; ^6^UK MS Register, Swansea Medical School, Swansea, UK

##### **Correspondence:** Paul Kamudoni (paul.kamudoni@merckgroup.com)

**Background**

A short form for assessing physical function in multiple sclerosis patients has recently been derived based on the PROMIS PF item bank. Strong content validity of the new short form was established based on two qualitative studies – involving concept elicitation and cognitive debriefing interviews.

**Objective**

The purpose of this analysis was to explore the dimensionality of the new PROMIS physical function short form in populations with relapsing and progressive forms of MS.

**Methods**

This study is a component of a multi-stage mixed-methods research program, including qualitative research with MS patients, expert panels (clinicians, measurement experts). A 52-week prospective longitudinal study including patients with MS, with EDSS score <7, is being carried out in the UK MS Register population. Participants are being assessed on the new short form and other PROs at baseline, week 1, week 25 and week 52. Item-level analyses, factor analyses, and item-response theory analyses were carried out to refine the instrument, at baseline. Data were analyzed using item-total correlations, exploratory factor analyses, confirmatory factor analyses and bifactor analyses, at baseline.

**Results**

Study participants (n =558) had a mean age of 49.9 (SD =9.7; range =19 to 65) years and 76% were female. Four items were removed from the short form, based on results from item level analyses, leaving 19 items. The mean (SD) T-score for the 19-item short form was 38.6 (SD =10.44; range =12.9 to 63.8). In the EFA, the first factor accounted for 66.4 % of the variance and the ratio of the first to second factor eigenvalues was 27:1. A CFA of one factor model for PROMIS Physical Function scores had excellent fit (Root Mean Square Error of Approximation [RMSEA] =0.0487, Tucker Lewis Index [TLI] =0.998, Confirmatory Fit Index [CFI] =0.998). A bifactor model showed Omega hierarchical of 0.97; while the explained common variance was 0.95.

**Conclusion**

The current results support unidimensionality of the new short form, which warrants a single overall physical function score as well as application of IRT-modelling approaches. On the other hand, the data showed limited measurement benefit from scoring of subdomains.

## P29. What are provider/patient PROMIS Scales as an assessment in primary care?

### Dan Kang^1^, Tyler Cuddeford^1^, Sarah Rahkola^2^, Jeff Houck^2^

#### ^1^George Fox University, Newberg, OR, USA; ^2^Providence Medical Group, Newberg, OR, USA

##### **Correspondence:** Dan Kang (dkang@georgefox.edu)

**Background**

A priority for primary care providers (PCP’s) is to address patient physical and mental symptoms. Current standard treatments often don’t address these symptoms requiring assistance from allied health providers (e.g. behavior & physical therapy). Yet, PCP’s may perceive new assessments as not helpful and worry about patient burden. **Objective:** The objective was to determine if PCP’s perceive Patient Reported Outcome Information System(PROMIS) scales helpful for standard assessment in primary care and do patients perceive these scales as difficult to complete?

**Methods**

PROMIS scales (fatigue, physical functions, pain interference, self-efficacy and depression) administered in the waiting room were collected on primary care patients for all diagnoses for 10 weeks. Knowledge tools were developed to communicate patient responses to the PCP at the point of care. PCP were shown patient scores prior to each patient visit. Subsequently, PCP providers ranked the helpfulness of the scales (0-10) and participated in qualitative interviews. Patients also ranked the difficulty of completing the scales (0-10). Chart review catalogued age, body mass index, comorbidities, and diagnosis.

**Results**

Average demographics indicated patients were older (66.9±17 years, n=369), of high body mass index (30.5±6.9, n=360) and several coexisting health problems (5.5±2.7, n=369). Visit diagnosis included annual (4.9%), cardiovascular (17.9%), endocrine (20.9%), musculoskeletal(17.9%) and other (38.0%). For 301 of 369 patients, 5 PCP’s on average ranked helpfulness as 7.9/10 (mode=8, median=8). For 66.8% interactions PCP’s ranked helpfulness as greater than 7/10. PCP’s indicated greater helpfulness on initial visits, complex diagnoses and requested specific treatments to address symptoms. Patients (348 of 369) on average ranked difficulty of completing scales as 1.9 (mode=1, median=1). A total of 74.8 % of patients ranked difficulty of completing scales as less than 3.

**Conclusions**

The knowledge tools and workflow were effective at the point of care, helping PCP’s prepare for their patient encounter. Patient difficulty in completing the scales was low. However, a barrier to implementation was the lack of specific, scalable, behavior/physical therapy treatments, to allay identified symptoms. PROMIS scales may provide a tool to effectively stratify, and measure outcomes, in patients with symptoms that may be responsive to behavior/physical therapy services.

## P30. The influence of mental health on patient-reported outcomes following anatomic total shoulder arthroplasty

### Eitan M. Kohan, Alexander W. Aleem, Aaron M. Chamberlain, Jay D. Keener

#### Washington University, Saint Louis, MO, USA

##### **Correspondence:** Eitan M. Kohan (emkohan@gmail.com)

**BACKGROUND**

Anxiety and depression symptoms have been shown to be associated with higher pain and lower functional scores in patients with symptomatic glenohumeral osteoarthritis. The influence of mental health on patient-reported outcomes following anatomic total shoulder arthroplasty (TSA) for glenohumeral osteoarthritis has not yet been fully investigated.

**METHODS**

This observational cohort study included 143 shoulders in 135 patients who underwent TSA for glenohumeral osteoarthritis. All patients completed preoperative and at least 1-year postoperative American Shoulder and Elbow Surgeons (ASES) score, Visual Analog Pain Scale (VAS), and Patient-Reported Outcome Measurement Information System (PROMIS) computer adaptive tests (CAT). The Western Ontario Osteoarthritis Score (WOOS) was collected with postoperative scores. PROMIS Anxiety and Depression scores were converted into Generalized Anxiety Disorder-7 and Patient Health Questionnaire-9 scores, respectively. Mean postoperative pain and functional scores, improvement from preoperative scores, and surgical regret were compared between varying severity of anxiety or depression.

**RESULTS**

Analysis showed that compared to patients with scores corresponding to no anxiety, patients with moderate-to-severe anxiety reported statistically significantly worse WOOS (465 vs. 227, *p*=.02) and PROMIS Upper Extremity (41.2 vs. 48.0, *p*=.03) and higher Pain Interference (PICAT) scores (53.3 vs. 45.8, *p*<.01) postoperatively. Compared to those without depression, those with moderate-to-severe depression reported worse postoperative ASES (77.3 vs 89.7, *p*=.02) and WOOS (463 vs 226, *p*=.01) and higher VAS (2.5 vs 1.8, *p*=.01) and PICAT (54.7 vs 46.5, *p*<.01). There were no significant differences in pre-to-postoperative improvement in any of the pain or functional outcome measures when comparing those with anxiety or depression and those without. Patients with moderate-to-severe depression were less likely to want to undergo the same procedure again (*p*=.03).

**CONCLUSIONS**

Patients with symptoms of depression and anxiety report improvement in pain and functional scores following TSA that is similar to those without depression or anxiety. Despite the similar improvement, those with moderate-to-severe depression and anxiety symptoms reported persistently lower functional and higher pain scores. Though most patients are satisfied following TSA, those with moderate-to-severe depression were more likely to regret undergoing surgery.

## O31. Estimating important differences for pediatric PROMIS measures in children with brain tumors

### Jin-Shei Lai^1^, Jennifer Beaumont^2^

#### ^1^Northwestern University, Chicago, IL, USA; ^2^Terasaki Research Institute, Los Angeles, CA, USA

##### **Correspondence:** Jin-Shei Lai (js-lai@northwestern.edu)

**Objective**

Despite improved survival rates in recent years, survivors of childhood brain tumors (BT) often experience detrimental, persistent health effects and treatment-related late effects across the lifespan. Pediatric PROMIS is a validated tool to monitor children’s symptom burden. The purpose of this study was to estimate important differences for pediatric PROMIS to assist clinicians/investigators in interpreting PROMIS T-scores.

**Methods**

Data from 464 participants (202 BT aged 8-21yrs and 262 parents of patients aged 5-21yrs) were used. Participants completed PROMIS measures and Symptom Distress Scale (SDS) (self- or proxy-reported versions). 223 (97 BT, 126 parents) completed the 12-month follow-up. Cross-sectional analyses compared baseline scores between groups (i.e., individual SDS items, parent-rated single QOL item, performance status, educational setting). Effect sizes (mean difference divided by the pooled standard deviation) were calculated to quantify the magnitude of group differences. For longitudinal analyses, patients were grouped into improved, unchanged, or worsened based on changes in SDS scores. The standardized response mean (SRM) was calculated by dividing the mean PROMIS change in a group by the standard deviation of the change scores. Anchor-based estimates should meet 3 criteria to be considered in the final important difference determination: 1) correlation > 0.3 between anchor and score; 2) sample size >10 in the change score group; 3) corresponding effect size or SRM between 0.2 and 0.8.

**Results**

Taking into account both cross-sectional and longitudinal analysis results, the estimated (minimally) important differences for parent-rated T-scores were: Anxiety 3-7 points, Depression, 3.5-6.5 points, Fatigue 4.5-7.5 points, Mobility 4-6 points, Peer Relationships 3-5 points, Upper Extremity 4-7.5 points, and Cognition 2.5 – 4.5 points. The estimated (minimally) important differences for child-rated T-scores were: Anxiety 4-6 points, Depression, 4-5 points, Fatigue 4.5-6.5 points, Mobility 3-6.5 points, Upper Extremity 2.5-8 points, and Cognition 2.5 – 5.5 points. None of the anchor-based estimates for child-rated Peer Relationships met the criteria for inclusion.

**Conclusions**

This study reported important differences of pediatric PROMIS on children with brain tumors. This information could be used for future studies to assist interpretation of intervention effectiveness.

## P33. The Importance of self-image for patients with adolescent idiopathic scoliosis: limitations of existing PROMIS domains

### J. Phillip Reynolds^1^, Reed Ling^1^, Liam Wong^1^, Madeleine Ball^1^, Yashar Javidan^1,2^, Eric Klineberg^1,2^, Rolando Roberto^1,2^

#### ^1^Shriners Hospitals for Children Northern California, Sacramento, CA, USA; ^2^University of California Davis Orthopaedic Surgery, Sacramento, CA, USA

##### **Correspondence:** J. Phillip Reynolds (preynolds@ucdavis.edu)

**Background**

Patient-Reported Outcomes Measurement Information System (PROMIS) is a series of well-validated quality-of-life questionnaires for children and adults with chronic medical conditions. Scoliosis Research Society-22r questionnaire (SRS-22r) is designed specifically for spinal deformity patients. The objectives of this study were to assess the ability of PROMIS and SRS-22r domains to detect differences between non-operative Adolescent Idiopathic Scoliosis (AIS) patients and operative patients at similar time points and to validate the utility of PROMIS in the pediatric spinal deformity population.

**Methods**

In this IRB-approved study, 91 subjects who completed both PROMIS and SRS-22r were retrospectively identified for study inclusion. The four PROMIS Short Form domains of interest include Mobility, Upper Extremity Function, Pain Interference, and Peer Relationship. At the same visit, the subjects also completed SRS function, pain, self-image, mental health, and satisfaction domains, with operative subjects completing all measures at their pre- and post-operative visits. Unpaired t-tests assessed differences between the operative and non-operative groups. We calculated Spearman rank correlation coefficients to evaluate the relationship between PROMIS and SRS-22r domains .

**Results**

91 subjects, 40 of whom underwent spinal surgical correction, were analyzed. None of the PROMIS domains significantly distinguished between operative and non-operative groups; however for SRS-22r, Self-Image was sensitive to differences between these groups (p=0.007). In regards to the validity of PROMIS, a moderate to strong correlation existed between several function and pain domains, with the strongest correlations between PROMIS Mobility and SRS-22r Function domain (r= 0.63, p < .01) and the PROMIS Pain Interference and SRS-22r Pain domain (r= -0.68, p < .01). The SRS-22r domains of Self-Image and Satisfaction poorly correlated with all tested PROMIS domains (r<0.40).

**Conclusions**

Our data demonstrates correlations between PROMIS and SRS-22r domains, corroborating previous investigations in patients with spinal deformity. However, there was a weak correlation between the PROMIS and SRS-22r domains of Self-Image and Satisfaction, which are clinically important in this patient population. Only the SRS-22r Self-Image domain was sensitive enough to determine significant differences between operative and non-operative groups. Development of PROMIS domains that report self-image and satisfaction for AIS should be developed and validated.

## O34. From statistician to clinician: The feedback of PROMIS® CATs within KLIK

### Maud M. van Muilekom^1^, Michiel A. J. Luijten^1,2^, Hedy A. van Oers^1^, Caroline B. Terwee^2^, Raphaële R.L. van Litsenburg^3,4^, Leo D. Roorda^5^, Martha A. Grootenhuis^3^, Lotte Haverman^1^

#### ^1^ Emma Children’s Hospital, Amsterdam UMC, University of Amsterdam, Psychosocial Department, Amsterdam, the Netherlands; ^2^ Amsterdam UMC, Vrije Universiteit, Epidemiology and Biostatistics, Amsterdam, the Netherlands; ^3^ Princess Máxima Center for Pediatric Oncology, Utrecht, the Netherlands; ^4^ Emma’s Children’s Hospital, Amsterdam UMC, Vrije Universiteit Amsterdam, Pediatric Oncology, Cancer Center Amsterdam, Amsterdam, the Netherlands; ^5^ Amsterdam Rehabilitation Research Center | Reade, Amsterdam, the Netherlands

##### **Correspondence:** Michiel A. J. Luijten (m.a.luijten@amc.uva.nl)

**Background**

KLIK is an evidence-based Patient Reported Outcome Measures (PROM) portal where patients and/or caregivers complete questionnaires about Health-Related Quality of Life (HRQOL), symptoms and psychosocial functioning. Answers are immediately converted into a KLIK ePROfile, which clinicians can discuss during consultation. Item responses and domain scores are most commonly fed back in traffic light colors and graphs, respectively. Currently PROMIS® item banks are implemented in KLIK, facilitating Computerized Adaptive Testing (CAT). New feedback options are required for CAT, as not all items are administered (estimates are computable) and PROMIS domain scores require different interpretation. This study aims to develop feedback options for PROMIS CATs within KLIK.

**Methods**

Focus groups were held with clinicians (pediatricians, psychologists, nurses, social workers, researchers) using KLIK. Literature-based feedback options were shown for individual items (i.e. item maps) and domain scores. Clinicians were asked about interpretability, comprehensibility, (color)design, and completeness of these options. Moreover, they were requested to describe their optimal feedback option. Data saturation was reached and data was analyzed using MaxQDA. A self-composed questionnaire will be send out to quantitatively assess clinicians’ preference regarding estimates of responses..

**Results**

In total, six focus groups were held (N=27 clinicians). According to clinicians, individual item feedback is necessary for using PROMs in clinical practice. Presenting the full item banks, with only responses (in traffic light colors) of administered items, was described as their optimal feedback option. Inclusion of response estimates of items that were not administered was considered difficult to interpret. Regarding domain score feedback, clinicians preferred graphs over textual options. In addition, they preferred separate graphs per domain, ranked in order of scores that were most alarming. Graphs should include normative lines (including standard deviation lines), traffic light colors and a well-defined y-axis (i.e., same directionality). There was disagreement about including numerical scores within graphs. Questionnaire results will be presented at the conference.

**Conclusions**

Overall, simplicity was considered most important when developing a new feedback method for PROMIS CATs. Once the questionnaires have been analyzed, we will, in collaboration with the Dutch-Flemish PROMIS National Center, design and subsequently evaluate the optimal feedback option to successfully implement PROMIS CATs in KLIK.

## P35. Validation of the pediatric Patient-Reported Outcomes Measurement Information System (PROMIS®) fatigue, sleep-related impairment and sleep disturbance item banks in the general Dutch population

### Shosha H.M. Peersmann^1,4^, Michiel A. J. Luijten^2,3^, Lotte Haverman^2^ ,Caroline B. Terwee^3^, Martha A. Grootenhuis^1^ , Raphaële R.L. van Litsenburg^1,4^

#### ^1^ Princess Máxima Center for Pediatric Oncology, Utrecht, the Netherlands; ^2^ Emma Children’s Hospital, Amsterdam UMC, University of Amsterdam, Psychosocial Department, Amsterdam, the Netherlands; ^3^ Amsterdam UMC, Vrije Universiteit, Epidemiology and Biostatistics, Amsterdam, the Netherlands; ^4^ Emma’s Children’s Hospital, Amsterdam UMC, Vrije Universiteit Amsterdam, Pediatric Oncology, Cancer Center Amsterdam, Amsterdam, the Netherlands

##### **Correspondence:** Michiel A. J. Luijten (m.a.luijten@amc.uva.nl)

**Background**

Our aim was to assess the validity and reliability of the pediatric v2.0 PROMIS Fatigue, v1.0 Sleep-Related Impairment and v1.0 Sleep Disturbance item banks in the general Dutch population and to provide normative data. Reliability of CATs and short-forms was also assessed.

**Methods**

Children 8-18 years old (n=1325), representative of the Dutch population on key demographics (age, sex, ethnicity, region, education level and social class), were asked to complete the PROMIS Fatigue, Sleep-Related Impairment and Sleep Disturbance item banks, consisting of 25, 13 and 15 items respectively. Unidimensionality was assessed using a CFA (CFI>0.95, TLI>0.95, RMSEA<0.10) and bi-factor analyses (ω_*h*_>0.80, explained common variance (ECV) >0.60). Local independence was assessed by looking at residual correlations (<0.20). Monotonicity was assessed using Mokken scale analyses (H_i_ >0.30 and scale H >0.50). A graded response model was fit to the data and the structural validity was assessed by looking at item-fit statistics (S-X^2^, p-value <0.001 indicates misfit). Reliability was calculated with the standard error of measurement (SEM). Amount of respondents with a reliable measurement (<0.32 SEM) and average SEM-value were compared between complete item banks, short-forms and CATs.

**Results**

The questionnaires were completed by 527 children (response rate of 39.7%). Unidimensionality was not supported for the Sleep Disturbance item bank (CFI=0.90, TLI=0.88, RMSEA=0.18, ω_*h*_=0.75, ECV=0.66). Assumptions were met for the remaining item banks. Concerning model fit, the Fatigue item bank contained two items (3224R1r & 4191R1r) and the Sleep-Related Impairment item bank one item (w026c), that did not fit the Dutch population, due to low discriminatory power of these items. Both item banks and short-forms measured reliably at the mean of the population and 2 SD in clinical relevant direction. CATs outperformed short-forms in terms of amount of reliably estimated respondents and test length.

**Conclusions**

The Fatigue and Sleep-Related Impairment item banks were successfully validated for use in the Dutch population, though certain items may not contribute to better measurements in the Dutch population and could be considered for removal in the Dutch versions of these item banks. Sleep Disturbance item bank requires further investigation into the cause of multidimensionality. Normative data are now available.

## P36. PROMIS forms demonstrate responsiveness in patients following arthroscopic rotator cuff repair across numerous health domains

### Felicity Fisk, Sreten Franovic, Joe Tramer, Noah Kuhlmann, Vasilios Moutzouros, Stephanie Muh, Eric Makhni

#### Henry Ford Health System, Detroit, MI, USA

##### **Correspondence:** Eric Makhni (ericmakhnimd@gmail.com)

**Background**

Recent studies have validated use of National Institutes of Health (NIH) Patient-Reported Outcomes Measurement Information System (PROMIS) health measures in patients with rotator cuff tear. These studies have demonstrated favorable administration and psychometric properties of PROMIS forms. However, the responsiveness of PROMIS computer adaptive test (CAT) forms in patients undergoing rotator cuff repair has not been investigated. The purpose of this study was to investigate the responsiveness of PROMIS CAT assessments post-operatively in patients undergoing arthroscopic rotator cuff repair.

**Methods**

All patients undergoing arthroscopic rotator cuff repair by one of three fellowship-trained surgeons were included in the study. PROMIS CAT upper extremity physical function ("PROMIS-UE"), pain interference ("PROMIS-PI"), and depression ("PROMIS-D") scores from pre-operative and 6-month post-operative visits were collected and analyzed. Patient-centric demographic factors, tear size, and biceps involvement were also correlated to pre- and post-operative PROMIS scores.

**Results**

A total of 101 patients were enrolled in the study. The average age was 59.8 ± 8.9 years with 51 males (50.5%). Pre-operative PROMIS-UE, PROMIS-PI and PROMIS-D CAT scores improved significantly from 29.8 ± 6.0, 62.6 ± 5.1, and 48.4 ± 8.7, respectively, to 40.9 ± 9.8, 51.2 ± 9.3, and 42.9 ± 9.0, respectively, at 6-month follow-up (p<0.001). Pre-operative correlations were found between PROMIS-UE and PROMIS-PI scores (p<.0.001) and between PROMIS-PI and PROMIS-D scores (p=0.001). No significant correlation was found between PROMIS-UE and PROMIS-D scores (p=0.08), pre-operatively. Pre-operative PROMIS-UE, PROMIS-PI or PROMIS-D scores were not correlated with rotator cuff tear size (p=0.4).

**Conclusions**

PROMIS CAT forms demonstrate responsiveness in patients undergoing arthroscopic rotator cuff repair across numerous domains.

## P37. Floor and ceiling effects of PROMIS in sports medicine patients undergoing non-operative and operative treatment

### Caleb M. Gulledge, D. Grace Smith, Vincent A. Lizzio, Alexander Ziedas, Stephanie J. Muh, Vasilios Moutzouros, Eric C. Makhni

#### Henry Ford Health System, Detroit, MI, USA

##### **Correspondence:** Eric C. Makhni (ericmakhnimd@gmail.com)

**Objective**

The Patient-Reported Outcomes Measurement Information System (PROMIS) computer adaptive tests (CAT) have emerged as an efficient technique for measuring patient-reported outcomes in orthopaedic patients. The purpose of this study was to investigate the floor and ceiling (F/C) effects of PROMIS CATs in patients presenting to a shoulder, elbow, and sports medicine orthopaedic clinic.

**Methods**

Patients prospectively completed PROMIS CATs, including physical function (PROMIS-PF), upper extremity function (PROMIS-UE), pain interference (PROMIS-PI), and depression (PROMIS-D), at their initial encounter and were retrospectively included in this study. Adult patients indicating a single complaint involving either the shoulder, knee, hip, or elbow were included. Patients were also grouped as either preoperative or nonoperative. F/C effects were defined as the proportion of respondents scoring the highest (ceiling) or lowest (floor) possible scores.

**Results**

3,460 patients were included (average age 50.1±17.0 years). PROMIS-PF demonstrated negligible F/C effects across knee and hip patients (≤0.2%). PROMIS-UE demonstrated negligible F/C effects in all shoulder patients (<2%; p=0.069-0.147), but displayed minor floor effects in preoperative elbow patients (7.1%; p=0.009) and minor ceiling effects in nonoperative elbow patients (6.9%; not different than preoperative elbow patients; 3.6%; p=0.378). PROMIS-PI displayed negligible F/C effects in all patients (<2%) except for minor floor effects in nonoperative elbow patients (6.3%; p<0.001). Finally, PROMIS-D displayed moderate to significant floor effects in all patient groups (12.7-34.7%). PROMIS-D had 0% ceiling effects in all groups.

**Conclusions**

The PROMIS-PF, PROMIS-UE and PROMIS-PI demonstrated generally favorable F/C effects for both nonoperative and preoperative patients. These findings justify consideration of PROMIS-PF, PROMIS-UE and PROMIS-PI CAT forms for clinical and research applications in shoulder, elbow, and sports medicine patients. While PROMIS-UE and PROMIS-PI demonstrated some minor F/C effects in elbow patients, these effects are within reasonable bounds and should not preclude them from further utilization. Additionally, we found moderate to significant floor effects for the PROMIS-D in all patient populations, which may be multifactorial in nature and limit its widespread utility.

## P38. Validating the adult PROMIS in pediatric sports medicine patients

### D. Grace Smith, Sreten Franovic, Caleb M. Gulledge, Eric C. Makhni

#### Henry Ford Health System, Detroit, MI, USA

##### **Correspondence:** Eric C. Makhni (ericmakhnimd@gmail.com)

**Objective**

The Patient-Reported Outcome Measurement Information System (PROMIS) has been validated in many different orthopaedic patient cohorts. However, the initial validation of PROMIS was performed in a cohort with a majority of older patients. Sports medicine physicians treat a diverse patient population with a significant portion of youth athletes; therefore, it is important that PROMIS scores are validated in the pediatric population. The purpose of this study was to validate adult PROMIS forms for use in a pediatric population.

**Methods**

116 patients (10-17 years old) presenting for a pre-operative clinic visit with one of two sports medicine orthopaedic surgeons were recruited for this study. Participants were asked to complete both the Pediatric and Adult PROMIS computer adaptive test (CAT) forms on an electronic tablet. The set of pediatric PROMIS CAT forms provided were Upper Extremity (“PROMIS-UE”), Mobility, Pain Interference (“PROMIS-PI”), and Depressive Symptoms (“PROMIS-Depressive Sx”). The corresponding adult forms were Upper Extremity (“PROMIS-UE”), Physical Function (“PROMIS-PF”), Pain Interference (“PROMIS-PI”), and Depression (“PROMIS-DE”). Mean and standard error values were compared between both groups, as well as correlations between respective adult and pediatric domains.

**Results**

Average±SD values for pediatric UE, PF, PI, and DE were 44.7±7.6, 37.1±8.4, 50.0±9.6, and 45.0±11.0, respectively. Average values for the adult domains were 41.6±8.9, 44.8±11.8, 55.8±7.5, and 44.6±9.7, respectively. Significant correlations were found among all four respective adult and pediatric domains: UE (r=0.400, p=0.032), PF (r=0.823, p<0.01), PI (r=0.778, p<0.01), and DE (r=0.862, p<0.01). Significant ceiling effects were found in the Pediatric UE and PF domains (14% and 5%), while none were found in the adult forms. Significant floor effects were noted in pediatric and adult PI and DE (10%, 25%, 8%, and 34%, respectively).

**Conclusions**

Adult PROMIS forms showed strong correlation with pediatric PROMIS-PF, PROMIS-PI, and PROMIS-DE. Both questionnaire sets demonstrated high floor effects in the PROMIS-DE form. Adult PROMIS assessments are suitable for use in the adult and pediatric sports medicine patient population.

## P39. Predicting fatigue in primary immunodeficiency: analysis of PROMIS fatigue from an immune deficiency foundation survey

### Rajiv Mallick^1^, Paul Bassett^2^, Tiffany Henderson^3^, Christopher Scalchunes^3^

#### ^1^CSL Behring, King of Prussia, PA, USA, ^2^Meridian HealthComms Ltd, Manchester, UK, ^3^Immune Deficiency Foundation, Towson, MD, USA

##### **Correspondence:** Rajiv Mallick (Rajiv.Mallick@cslbehring.com)

**Objectives**

To characterize predictors of fatigue in patients with primary immunodeficiency (PID) receiving subcutaneous immunoglobulin (SCIG); in turn, to evaluate the impact of fatigue on general health perception (GHP).

**Methods**

We evaluated Immune Deficiency Foundation (IDF) survey data of patient-reported treatment experiences (infusions and self-administration training), fatigue, and GHP. Fatigue was assessed using the PROMIS Fatigue Short Form 7a (SF-7a) and Parent/Caregiver Proxy SF-10 respectively (0= least fatigue; 100 most fatigue). GHP was assessed as an anchored numeric rating scale (1= poor health; 7= excellent). Univariate analyses evaluated categorical associations between PROMIS fatigue T-scores and GHP score tertiles (fatigue T-score tertiles: ‘low’ ≤53, ‘middle’ >53 to <61, ‘high’ ≥61–85; GHP: ‘high’, 6–7, ‘middle’ 5, ‘low’ ≤4) with SCIG infusion and training categories. Multivariate logistic regression analyses identified predictors of low fatigue (defined as placement in the lowest (best) tertile for fatigue) and high GHP scores. Independent predictors were finalized by backward selection of significant covariates from the univariate analyses.

**Results**

Of 366 SCIG respondents with PID (326 adults; 40 parent/caregivers); the mean (standard deviation [SD]) PROMIS fatigue T-scores were 57.1 (8.5) and 55.0 (12.5) for adults and parent/caregivers respectively. Being ‘very confident’ after SCIG training, actual infusion time <2 hours, and total infusion time (including preparation time) <3 hours were associated with significantly higher probabilities (chi-square p<0.05) of low fatigue. Adjusting for all other covariates, including history on overall immunoglobulin therapy in general and SCIG therapy in particular, being ‘very confident’ after SCIG training (Odds Ratio [OR] = 1.95 [95% CI, 1.16–3.28]) and having an actual infusion time < 2 hours (OR = 1.80 [1.14–2.82]) were associated with almost double the odds of low fatigue. Being in the best tertile of PROMIS fatigue T-scores predicted 8 times higher odds (OR = 8.26 [4.56–15.0]) of a high GHP score.

**Conclusions**

Although self-reported patient fatigue in PID is multi-factorial, our results suggest that facilitating easier IG treatment by (a) ensuring patients are trained on SCIG self-administration to a high level of confidence and (b) enabling shorter self-infusion times may be associated with significant improvement in fatigue to near normal population levels.

**Funding:** CSL Behring sponsored the study

## P40. Survey delivery windows to reduce respondent burden in clinical PRO collections

### Allison W. McIntyre, Kathleen Fear, Daniel Hudy

#### University of Rochester, Rochester, NY, USA

##### **Correspondence:** Allison W. McIntyre (allisonw_mcintyre@urmc.rochester.edu)

**Background**

The clinical collection of patient reported outcomes (PRO) is essential as providers, payers and quality programs learn how the patient voice contributes to treatment plans and satisfaction. When a patient is allowed to self-report their biopsychosocial health status using the Patient Reported Measurement Information System (PROMIS), misinterpretation is unlikely and engagement and communication improve.

Ideally, a clinician would ask many PRO, as often as possible, to develop a full picture of each patient. However, that can result in the consequences of respondent burden, including incomplete surveys and dissatisfaction. Long survey length and emotionally stressful questions are obvious contributors, however, frequency of administration can be just as burdensome.

Delivery windows allow the assignment of tailored administration intervals to individual PROMIS instruments. They are especially important when multiple provider visits are required and the collection of PRO data at each visit may not provide added value to the patient or provider.

**Methods**

Using observational data, administration statistics and un-structured interviews, the implementation team collected information from stakeholders in a busy ambulatory Physical Therapy practice on the timing of instrument administration, which was originally set to every patient, every visit.

Consensus was sought on the length of delivery window, which was expanded to three weeks. Administration and other data was then compared for two months prior and after initiation of the new delivery window.

**Results**

The three week delivery window captures relevant data from regularly scheduled PT status visits and minimizes respondent burden by one-third for patients with weekly PT appointments. The administration rate increased by 19.2% and the number of evaluations in which patients completed all instruments increased by 12.5%. Check-in staff burden is also reduced, as they no longer need to make decisions about which patients need PRO administered; the PROMIS collection platform automatically administers PRO only as needed.

**Conclusions**

The lure of a profusion of PRO data must be checked by the reality that patients, and the data they provide, can be affected by the demands placed upon them. Increasing time between administrations has had a positive effect on both the administration and completion of PROMIS data in this busy PT clinic.

## P41. How to best display PROMIS Global-10 data in the clinic: perspectives from primary care physicians

### Danny Mou, Elena Cavallo, Marilyn Heng, Rachel Sisodia

#### Massachusetts General Hospital Physician Organization, Boston, MA, USA

##### **Correspondence:** Danny Mou (dmou2@partners.org)

**Objective**

To assess primary care physician’s perspective of how to display the PROMIS Global-10 data in a way that optimizes clinical relevance

**Methods**

We conducted a literature review of best practices in displaying data for clinicians. We then used principles from this primary literature to develop a series of sample displays of PROMIS Global-10 data. We conducted semi-structured interviews with nine primary care physicians (PCPs) at a large academic medical center to elicit opinions of these sample displays, and incorporated their suggestions in an iterative process as the interviews progressed.

**Results**

Feedback from the PCPs varied widely. Positive responses for our PROMIS Global-10 displays include the ability to trend symptoms over time, the nuances provided by a score rather than binary responses, and the relevance of the Activities of Daily Living assessment. Critiques of the PROMIS Global-10 displays include questionable clinical relevance, difficult to interpret data, and questions that are too generalized. 2 PCPs requested that these images be able to be pulled in the electronic health record (EHR) note. It was repeatedly emphasized that the data should be up to date and easy to access at the time of clinic visit. Data display principles used included consistent axes directions (i.e., higher on axes always means better health), visual aids (i.e., happy and sad faces on axes), and color coding.

**Conclusions**

In order to optimize the relevance of PROMIS Global-10 in the clinical setting, we must proactively understand the perspective of the clinician end-user. Data display is a critical factor in clinician adoption and user experience. We have preliminarily shown that there is a wide range of opinions from PCPs on how PROMIS Global-10 data should be displayed. More interviews and surveys should be conducted to further define the clinicians’ perspectives.

## P42. Validation of the PROMIS Preference scoring system (PROPr) in patients with End-Stage Kidney Disease (ESKD)

### Jing Zhang^1^, Daniel Breitner^1^, Barry Dewitt^2^, Janel Hanmer^3^, Mohammed Saqib^1^, Dan Li^1^, Nathaniel Edwards^1^, John Peipert^4^, Marta Novak^5^, Istvan Mucsi^1^

#### ^1^ Multi-Organ Transplant Program, University Health Network and University of Toronto, Toronto, Canada; ^2^ Department of Engineering & Public Policy, Carnegie Mellon University, Pittsburgh, PA, USA; ^3^ Department of General Internal Medicine, University of Pittsburgh Medical Center, Pittsburgh, PA, USA; ^4^ Department of Medical Social Sciences, Northwestern University, Chicago, IL, USA; ^5^ Centre for Mental Health, University Health Network, Toronto, ON, Canada

##### **Correspondence:** Istvan Mucsi (istvan.mucsi@utoronto.ca)

**Background**

The PROMIS Preference (PROPr) score is a preference-based summary score within the Patient-Reported Outcomes Measurement Information System (PROMIS) that assigns values to health states. We assessed the construct validity (convergent and known-groups) of PROPr among patients with ESKD and compared PROPr with the EQ5D5L and SF6D preference-based measures.

**Methods**

A cross-sectional sample of adults with ESKD (on dialysis and kidney transplant recipients [KTR]) completed questionnaires including PROMIS57 (7 domains: anxiety, depression, fatigue, physical function, sleep disturbance, pain interference and ability to participate in social roles), Patient Health Questionnaire (PHQ9), Edmonton Symptom Assessment Scale revised (ESASr), Kidney Disease Quality of Life-36 (KDQOL36), and EQ5D5L. The SF6D was generated from the SF12 (part of KDQOL36). PROPr is estimated from the PROMIS57 domain scores. The final score ranges from -0.022 (all-worst state) to 1.0 (full health). Known-group comparisons were evaluated using age- and sex-stratified median scores and calculating “health condition impact estimates”, that is the coefficient for a health condition when a summary score was regressed on age, gender, and a single health condition using ordinary least squares regression. Convergent validity was assessed with Pearson correlation between PROPr and other preference summary scores.

**Results**

Mean (SD) age of the 318 participants was 58 (17) years, 57% were male and 51% Caucasian. Median (IQR) scores were 0.38 (0.22-0.61), 0.71 (0.58-0.86) and 0.85 (0.67-0.91) for PROPr, SF6D and EQ5D5L, respectively. PROPr and SF6D scores were less subject to ceiling effects compared to EQ5D5L. All utility measures were associated with the clinical conditions assessed. The age and sex adjusted condition impact was larger for PROPr for all conditions tested compared to the other two scores. Condition impact for PROPr was: KTR vs. dialysis (-0.21, P<0.001), low vs. high comorbidity (-0.10, P<0.001), and low vs. high depression (-0.31, P<0.001). Strong correlations were observed between PROPr and EQ5D5L (rho=0.67) and SF6D (rho=0.74).

**Conclusions**

These results provide evidence of the PROPr’s validity among patients with ESKD. Moreover, PROPr may be more sensitive to differences in health states compared to other preference-based measures.

## O43. Development of a clinic implementation roadmap for the Epic PROMIS app

### Therese A. Nelson^1^, Jiang Bian^3^, Andrew D. Boyd^4^, Bhrandon A. Harris^4^, Kelly Hynes^2^, Karl Kochendorfer^4^, David Liebovitz^2^, Kayla Martin^3^, Donald Weinbrenner^3^, Sonya H. White^3^, Nan E. Rothrock^1^, Annette L. Valenta^4^, Justin B. Starren^1^

#### ^1^Northwestern University, Chicago, IL, USA; ^2^University of Chicago, Chicago, IL USA; ^3^University of Florida, Gainesville, FL, USA; ^4^University of Illinois at Chicago, Chicago, IL, USA

##### **Correspondence**: Therese A. Nelson (therese.nelson@northwestern.edu)

**Background**

To implement the Epic PROMIS app successfully in a clinical setting, a clinic and an institution must make many collaborative and highly consequential decisions and arrangements. These include defining clinical goals; selecting PROMIS measures; determining optimal populations, triggers, workflows, technical resources, and results management; and weighing institutional priorities and requirements. This presentation will present a roadmap to PROMIS Implementation and discuss results in four clinics at three initial sites.

**Methods**

The EHR Access to Seamless Integration of PROMIS (EASI-PRO) consortium consists of nine universities sharing the goal of integrating PROMIS into electronic health records (EHRs). Four EASI-PRO sites worked together to develop a roadmap that clinics can use to prepare for Epic PROMIS. To begin, information from various sources, including the HealthMeasures website, workshops and papers on PRO integration, was assembled and clustered thematically into an Implementation Guide that incorporates sociotechnical factors from the Human-Organization-Technology Fit (HOT-fit; Yusof et al., 2008) framework. Content from the Implementation Guide was transformed into an Implementation Survey consisting of 95 discrete fields, which each team completed through semi-structured interviews and collaboration among key stakeholders, including clinicians, informaticians, and PRO measurement scientists. Surveys were summarized into Clinic Implementation Plans.

**Results**

Salient themes in our results include the critical role of the physician champion in providing medical and clinical leadership, the importance of working with multiple partners, and the need to customize the PROMIS app to meet clinical aims. As an example of customization, an orthopedic clinic scheduled assessments of key variables at pre-determined timepoints; whereas a geriatrics clinic synchronized assessments with appointments to ensure prompt clinician attention. Workflow issues required the most tailoring by site and involved financial, technical, cultural and practical considerations. Implementation required both a step-wise and an iterative approach.

**Conclusions**

Our project elucidated the many local factors that are highly consequential in the success of a PROMIS clinical implementation. We produced an Implementation Guide and an Implementation Survey to facilitate integration of PROs in clinical practice. Along with four real-world Clinic Implementation Plans, these materials constitute a Clinic Implementation Roadmap that is available for use by future clinics wishing to implement PROMIS.

## O44. Feasibility of using PROMIS-CAT to capture patient reported outcomes in neurosurgery outpatient setting

### Mark Nyman^1^, Kelsey Wolff^2^, Anshit Goyal^3,4^, Mohammed Ali Alvi^3,4^,Sandy Goncalves^3,4^, Travis Paul^3^, Aaron Biedermann^3^, Carolyn M. Macken^2^, Janine Kamath^2^, Andrea Cheville^4^, Mohamad Bydon^3,4^

#### ^1^Department of Internal Medicine, Rochester, MN, USA; ^2^Management Engineering and Internal Consulting, Mayo Clinic, Rochester, MN, USA; ^3^Mayo Clinic Neuro-Informatics Laboratory, Mayo Clinic, Rochester, MN, USA; ^4^Department of Neurologic Surgery, Mayo Clinic, Rochester, MN, USA; ^5^Department of Physical Medicine and Rehabilitation, Mayo Clinic, Rochester, MN, USA

##### **Correspondence**: Mark Nyman (nyman.mark@mayo.edu)

**Background**

Current challenges around Patient Reported Outcomes (PRO) administration include redundant and uncoordinated PRO collection leading to excessive patient burden and diminished response rates. In this pilot, we aimed to improve the completion rate of baseline Patient-Reported Outcomes Measurement Information System (PROMIS) questionnaires among patients presenting for outpatient evaluation in Neurologic Surgery, while reducing patient burden, using a computerized adaptive testing (CAT) format.

**Methods**

PROMIS-CAT was selected as the tool for capturing PROs using our institutional patient online services (POS) portal, linked directly to the electronic health record (EHR). Prior to the pilot, PROMIS-29 was administered online via an iPad at the point-of-care immediately prior to provider encounters, but was not immediately available within the EHR. The switch to PROMIS-CAT was driven by the benefits of enhanced efficiency and precision with CAT, as well as the immediate availability of PRO data in the EHR for provider reference. With PROMIS-CAT, participant responses inform a computer algorithm to select the subsequent items from an item bank that are most likely to inform trait estimation.

**Results**

Prior to the pilot, PROMIS-29 completion rate was 30%. During the two month pilot, 1863 patients were assigned PROMIS-CAT, of which 1285 or 69% of patients completed the questionnaire. The average number of questions answered by each patient was 45.2. Upper Extremity Function (which is not included as a domain within PROMIS-29) represented the domain with the highest number of questions administered while the least number of questions were administered in Fatigue and Physical Function domains.

**Conclusions**

EHR linked CAT may represent a valid tool to increase PRO collection rates. Selective domain administration may alleviate patient burden. Use of a cross-cutting PRO like PROMIS-CAT over multiple redundant legacy PRO measures, may also alleviate patient burden.

## P45. Correlates of informational and emotional social support among Transition Age Youth (TAY) probationers exiting San Diego county jail and who attend the UCSD RELINK program

### Sarah Hiller-Venegas, Tamara D. Parker, Maurice Lyles, Emily Berliant, Cielo Jimenez, Zephon Lister, Todd Edwards, Sarah Linke, Victoria D. Ojeda

#### University of California San Diego School of Medicine Department of Family Medicine and Public Health, San Diego, CA, USA

##### **Correspondence**: Victoria D. Ojeda (vojeda@ucsd.edu)

**Background**

Transition age youth (TAY) reentrants face challenges to (re-)establishing informational (i.e., advice on how to navigate life) and emotional (i.e., caring, supportive individuals) social support relationships. This analysis describes correlates of informational/emotional social support among probationers age 18-26 enrolled in the UCSD RELINK program, which aims to reduce racial/ethnic disparities in health status/access to care by improving linkages to health/social services, and health/wellness-related knowledge/skills/behaviors.

**Methods**

From 2017-2019, 90 participants completed baseline questionnaires. We adapted Berkman et al.’s “Social Networks and Health” framework to identify factors that may influence or be influenced by social support. Univariate and multivariable logistic regression identified factors significantly associated with social support scores, measured using four PROMIS informational and emotional social support short scale items. Covariates were measured using validated measures (e.g., PHQ9, GAD2, ACES).

**Results**

Between 34-44% of participants indicated “never/rarely/sometimes” (vs. “usually/always”) on items indicating the availability of informational support, while 28-39% indicated the same for the emotional support items. Factors significantly (p<0.05) associated with lower social support scores in univariate analyses included older age (e.g., age 24-26 vs. age 18-20), parenting or being pregnant, annual income ≤$20,000, food insecurity, and multiple mental health outcome measures including moderate-severe depressive symptoms, positive anxiety symptom screener, reporting ≥4 adverse childhood events, and self-reported need of mental health care. Higher scores on items reflecting resilience and self-esteem were significantly associated with higher social support scores. Parenting status, low income, and depressive symptoms were among factors chosen for the final model that retained their significant association with social support.

**Conclusions**

This pilot study found that many TAY probationers reported deficits in informational/emotional support. Findings regarding low income, mental health outcomes, and resilience are reflected in similar studies, and point to the vulnerability perpetuated by a lack of social support. Pregnant/parenting TAY may feel especially isolated due to life changes, or because their current social support is insufficient given their increased need compared to non-parenting TAY. Public health interventions that provide social support or help participants to build social skills/networks may buffer the impact of these correlated factors on overall wellbeing.

## P46. Spanish translation and linguistic validation of PROMIS® sexual function and satisfaction measures: challenges and solutions

### Barbara Perez^1^, Benjamin Arnold^1^, Emily Parks-Vernizzi^1^, Helena Correia^2^

#### ^1^FACITtrans, Ponte Vedra, FL, USA; ^2^Northwestern University Feinberg School of Medicine, Department of Medical Social Sciences, Chicago, IL USA

##### **Correspondence**: Emily Parks-Vernizzi (Emily Parks-Vernizzi; eparks@facit.org)

**Objective**

The Patient Reported Outcomes Measurement Information System Sexual Function and Satisfaction (PROMIS SexFS) items measure a range of sexual activities, symptoms, functioning, and evaluation of experiences over the past 30 days. The purpose of this study was to translate and linguistically validate the PROMIS SexFS measures into Universal Spanish and to present and discuss the challenges and solutions encountered throughout the work.

**Methods**

The PROMIS SexFS items were translated based on the FACIT methodology: two forward translations, one reconciled version of the two forward translations, one back-translation into English, independent reviews, formatting and proofreading. Spanish-speaking participants in the USA and Mexico completed the questionnaires and participated in cognitive interviews to assess the relevance, understandability, and appropriateness of the translations. Qualitative analyses of participants’ comments were used to assess the conceptual equivalence of each translated version.

**Results**

The study sample consisted of 33 native Spanish-speaking participants (18 males/15 females); 13 were interviewed in Mexico, and 20 were interviewed in the USA. All participants reported themselves as sexually active. The mean age was 37.8 (25-58) years. Examples of terms that proved challenging to translate are “hot flashes” and “hair loss.” Two terms were used in the translation of “hot flashes”: “calores repentinos” (sudden heat waves) and “sofocos” (hot flashes). During translation, linguists confirmed the use of “sofocos” in many countries to describe “hot flashes”. The addition of “calores repentinos” seemed necessary as a more descriptive term. When translating “hair loss,” linguists discussed the distinction between hair on the body (vello) versus on the head (cabello). Additionally, the more idiomatic translation of “loss” in this context “falling” (“caída”) was chosen over the literal translation of “loss” (“pérdida”). Developer feedback confirmed a preference for “hair loss on the head or body.” During cognitive interviews, the translations for “hot flashes” (calores repentinos y sofocos) and “hair loss” (la caída de cabello o vello) were endorsed by participants.

**Conclusions**

The Universal Spanish version of the PROMIS SexFS items is conceptually equivalent to the English source and considered acceptable for patient-reported outcomes assessment in international research and clinical trials.

## O47. Development of physical functioning items for PROMIS® to be used with minority elderly

### Sylvia H. Paz, Ron D. Hays

#### UCLA Division of General Internal Medicine & Health Services Research, Department of Medicine, Los Angeles, CA, USA

##### **Correspondence**: Sylvia H. Paz (shpaz@ucla.edu)

**Background**

Physical functioning is an important health domain for the elderly and one of the strongest predictors of health care utilization and mortality. The purpose of this study is to develop physical functioning items appropriate for the elderly that can be added to the Patient Reported Outcomes Measurement Information System (PROMIS®) Physical Function item bank.

**Methods**

Six focus groups of elderly subjects belonging to different minority groups and two focus groups with non-Hispanic White elderly were conducted. Twenty-four cognitive interviews with the same ethnic groups evaluating the revised and new items were conducted. Focus groups and cognitive interviews took place in five locations in South, South-Central, and North-East Los Angeles, CA.

**Results**

Mean age of focus group participants was 76 years (range 65-91), 54% were female, 31% African-American, 25% Spanish-speaking Latinos, 9% English-speaking Latinos, and 35% were non-Hispanic Whites. The cognitive interviews included 12 participants who were 65-74, 10 75-84, and 2 85 or older; 66% were female; 42% Hispanic or Latino; 29% Spanish-speaking Latinos; 38% African American; and 50% were White.

Many of the PROMIS Physical Functioning items were not relevant to the minority focus group participants. Some items had confusing wording and were lengthy. In addition, participants were often unsure whether to include their use of physical aids when responding to items. Fewer response errors were observed among the non-Hispanic white focus group participants.

Cognitive Interviews revealed that some of the revised items were clearer and preferred to the original items. Revised wording about the use of different physical aids reduced respondent confusion.

**Conclusions**

Problems with item wording, examples used, how to respond if using aids, and response options may affect the reliability and validity of the PROMIS Physical Function measure among minority elderly. New items have been developed and adapted to ensure they are relevant to elderly minorities. These items will be administered along with existing PROMIS physical functioning items in future studies to evaluate their psychometric properties.

**Acknowledgements**

Drs. Paz and Hays received support from the University of California, Los Angeles (UCLA), Resource Centers for Minority Aging Research Center for Health Improvement of Minority Elderly (RCMAR/CHIME) under NIH/NIA Grant P30-AG021684, and received support from the UCLA Clinical and Translational Science Institute (CTSI) under NIH/NCATS Grant Number UL1TR001881.

## O48. Comparison of health-related quality of life among kidney and liver transplant patients using the PROMIS global health scale

### John Devin Peipert^1, 2^, Amy Waterman^3^, Farrukh Koraishy^4^, Meeta Evers^5^, Henry Randall^5^, Mark Schnitzler^5^, Krista Lentine^4, 5^

#### ^1^Department of Medical Social Sciences, Northwestern University Feinberg School of Medicine, Chicago, IL, USA; ^2^Northwestern University Transplant Outcomes Research Collaborative, Comprehensive Transplant Center, Feinberg School of Medicine, Chicago, IL, USA; ^3^ Transplant Research and Education Center, Division of Nephrology, UCLA Geffen School of Medicine, Los Angeles, CA, USA; ^4^ Department of Medicine, Saint Louis University School of Medicine, St. Louis, MO, USA; ^5^ Saint Louis University Center for Abdominal Transplantation, Saint Louis University School of Medicine, St. Louis, MO, USA

##### **Correspondence**: John Devin Peipert (john.peipert@northwestern.edu)

**Background**

Survival after kidney transplant (KT) and liver transplant (LT) are increasing, turning attention to health-related quality of life (HRQOL) among these patients. Brief but valid measures are needed to screen HRQOL among KT and LT patients. In this study, we examined and compared the measurement properties of the 10-item PROMIS Global Health Scale (GHS) pre- and post-transplant among KT and LT patients.

**Methods**

Data were from KT and LT patients at a single transplant center in the United States. PROMIS GHS was assessed pre-transplant (KT, n=189; LT, n=88) and 6 months post-transplant (KT, n=43; LT, n=16). We estimated global physical health (GPH) and global mental health (GMH) summary scores from the GHS. We compared KT and LT to the US general population normative mean value of 50 (standard deviation = 10). We examined the dimensionality of the PROMIS GHS with bifactor exploratory factor analysis (bEFA). We then estimated associations between PROMIS GPH and GMH scores with clinician-rated functional status sourced from the Scientific Registry for Transplant Recipients.

**Results**

Among KT patients, the mean GPH and GMH scores at pre-transplant were 46.3 and 50.2, respectively, which increased to 51.1 and 54.1 at 6 mo post-transplant. Among LT patients, mean GPH and GMH scores at pre-transplant were 42.1 and 46.3, respectively, which increased to 44.7 and 50.1 at 6 mo post-transplant. For both KT and LT, unlike previous analyses for the GHS, bEFA suggested unidimensionality with a strong general factor and 3 local factors instead of two factors representing the GPH and GMH. Omega hierarchical estimates were 0.69 for KT patients and 0.70 for LT patients, and larger loadings on the general vs. local factors were observed for most items. Pre-transplant differences in functional status were not statistically significant for KT patients. However, in comparison to LT patients with normal function, those unable to carry-on normal activities had significantly lower mean GPH (39.9 vs. 47.4, p<0.001) and GMH (44.5 vs. 50.3, p=0.01) scores.

**Conclusions**

The PROMIS GPH indicated sensitivity to health in KT and LT patients, reflecting significantly lower health pre-transplant that improved post-transplant. Despite this, dimensionality requires further examination.

## P49. PROMIS measures capture negative impact of familial chylomicronemia syndrome on health-related quality of life

### Rina S. Fox^1^, John Devin Peipert^1^, Glenn Phillips^2^,Stuart Hurst^3^, Dave Cella^1^

#### ^1^Department of Medical Social Sciences, Northwestern University Feinberg School of Medicine, Chicago, IL, USA; ^2^Rhythm Pharmaceuticals, Boston, MA, USA; ^3^Akcea Pharmaceuticals, Boston, MA, USA

##### **Correspondence**: John Devin Peipert (john.peipert@northwestern.edu)

**Background**

Familial Chylomicronemia Syndrome (FCS) is a rare genetic disorder wherein the body does not break down fats appropriately. FCS significantly impacts patients’ physical, mental, and social health. Legacy patient-reported outcome (PRO) measures are not sensitive to FCS’s health impact. NIH PROMIS® measures may be more sensitive to capturing health-related quality of life (HRQOL) in FCS patients. The objective of this project was to assess a broad range of PROMIS measures covering physical, mental, and social HRQOL to determine their suitability for the FCS population.

**Methods**

Adult FCS patients living in the United States (*N*=25) were administered several PROMIS short forms: global health, 57- item profile (including depression, anxiety, physical function, pain interference, fatigue, sleep disturbance, and ability to participate in social roles and activities), cognitive function, self-efficacy for managing social interactions, self-efficacy for managing symptoms, gastrointestinal belly pain, and social isolation. PROMIS measures are calibrated to have a mean T-score of 50 (*SD*=10), with the sleep disturbance and self-efficacy for managing social interactions scales normed to other chronic illness populations, and all other scales normed to the United States general population. Across measures higher scores indicate more of the construct assessed. Descriptive statistics were calculated for each measure.

**Results**

Scores were more than 0.5 standard deviations (SD) worse than the PROMIS reference group, indicating reduced global physical (T=42.69) and mental health (T=43.38), reduced physical function (T=44.16), and lower self-efficacy for managing social interactions (T=44.74). Scores also demonstrated elevated anxiety (T=57.16), depression (T=55.70), fatigue (T=57.41), pain interference (T=56.92), and belly pain (T=55.24). Sleep disturbance (T=58.14) and cognitive function (T=41.60) deficits were closer to a full SD worse than the PROMIS reference group. Responses at the floor were observed for 10 of the 16 administered tests and responses at the ceiling were observed for seven of the 16 administered tests.

**Conclusions**

Results support of the sensitivity of PROMIS measures among patients with FCS. PROMIS measures capture the functional impact and symptom burden associated with FCS, and the broad range of symptom severity experienced by patients with FCS.

## P50. Does health assessment using PROMIS scales enhance clinical decision making for patients attending physical therapy?

### Li-Zandre Philbrook, Ryan Jacobson, Dan Kang, Tyler Cuddeford, Jeff Houck

#### George Fox University, Newberg, OR, USA

##### **Correspondence**: Li-Zandre Philbrook (lphilbrook@georgefox.edu)

**Background**

Which patient reported outcomes capture symptoms important to patients and providers at the initial assessment is undetermined. The proportion of patients presenting with symptoms and convergent validity among scales is a good initial assessment of which symptoms to target to serve patients.

**Objective**

To describe the proportion of patients that identify one of four generic symptom scales (Patient-Reported Outcomes Measurement Information System (PROMIS)) as their primary health problem at their initial visit to physical therapy and to determine the convergent validity of fatigue and self-efficacy of symptom management (SE) with more typical scales: physical function (PF) and pain interference (PI).

**Methods**

The PROMIS scales are routinely administered clinically. A total of 115 physical therapy clients were on average 43.0 (20.2) y.o., had a body mass index of 26.1 (8.3) and 65.2% were female. Problems in order of prevalence were: spine 54.8%, lower extremity 29.6%, upper extremity 13.9% and other 1.7%. All PROMIS scales were coded by 0.5 standard deviation increments, where negative values were worse than normal and positive values were better than normal. Symptoms were categorized: greater than 1 standard deviation (SD) worse than normal = moderate/severe; 0.5-1.0 SD = mild, and 0.5 worse than normal or better were considered with in normal limits (WNL). The primary symptom categories included: WNL, PForPI(mild-severe), PForPI(mild-severe)+SE(mild-severe), PForPI(mild-severe)+Fatigue(mild-severe), PForPI(mild-severe)+SE(mild-severe)+Fatigue(mild-severe). Strong convergence was considered greater than 0.7 using spearman correlations.

**Results**

The proportion of patients with mild or worse symptoms per scale was: PI 63.4%, PF 53.1%, Fatigue 42.6%, and SE 40%. The primary symptom categories were as follows: WNL 21.7%, PForPI 21.7%, PForPI+SE 16.5%, PForPI+Fatigue 27%, PForPI+SE+Fatigue 13%. Except for PF and PI (pho=-0.76), significant correlations were lower than 0.7 among scales, suggesting SE and Fatigue vary sufficiently to warrant separate assessment.

**Conclusions**

The most prevalent symptoms were PI and PF however these symptoms frequently combined with SE and Fatigue. The primary symptom severity varied considerably with fatigue (27%) and self-efficacy (16.5%) or both (13%). The lack of strong convergence of SE and Fatigue with PF/PI suggests these symptoms warrant separate assessment. Fatigue and SE symptoms occur frequently enough to warrant specific treatment.

## O51. Adolescent Idiopathic Scoliosis patients with pain have lower pre- and post-operative Patient Reported Outcomes Measurement Information System (PROMIS) domain scores compared to their non-painful peers

### Reed Ling^1^, J. Phillip Reynolds^2,^, Liam Wong^1,^, Yashar Javidan^1,2^, Eric O. Klineberg^1,2^, Rolando F. Roberto^1,2^

#### ^1^Shriners Hospitals for Children Northern California, Sacramento, CA, USA; ^2^University of California Davis School of Medicine, Sacramento, CA, USA

##### **Correspondence**: J. Phillip Reynolds (preynolds@ucdavis.edu)

**Background**

Approximately one-third of patients with Adolescent Idiopathic Scoliosis (AIS) have pain at the time of diagnosis. Prior studies have shown that there is significant improvement in postoperative SRS-22r scores; however, quality of life measures may remain lower than those with non-painful AIS. The purpose of this study was to: 1) investigate changes in pain scores in preoperative and postoperative AIS patients using the PROMIS Pain Interference metric, and 2) assess the relationship between pain response and severity of pediatric spinal deformity.

**Methods**

This IRB-approved retrospective study includes 263 AIS patients who underwent corrective spinal surgery. Four PROMIS domains (Mobility, Upper Extremity Function (UE), Pain Interference (PI), and Peer Relationship) were administered to a cohort of 18 patients at preoperative and early postoperative visits (6 to 12 weeks). Nonpainful and Elevated preoperative pain groups were categorized using the PROMIS-PI domain. Fischer’s exact test and Student t-tests were used to detect differences between curve magnitude and to explore differences across PROMIS domain scores by pain levels and follow-up intervals.

**Results**

There are significant differences between the preoperative pain groups and Mobility, UE and PI (p<0.01 for all). Patients with elevated pain had worse UE scores at baseline and at the 12 week postoperative follow-up. (P<0.01). The nonpainful group had a significantly larger decrease in Mobility and UE from the preoperative and postoperative visit (p<0.01) compared to their elevated pain peers. Curve magnitude did not predict preoperative pain as both groups had statistically similar average cobb (70° vs 73°, p=.96), although the nonpainful group had a greater percentage of curves ≥70° (66% vs 33%).

**Conclusions**

PROMIS is a useful tool for quantifying patient-reported outcomes in children with AIS. Our early data suggests that patients with increased pain demonstrate significantly lower physical function scores at baseline and 12 weeks postoperatively despite having similar preoperative curves. The nonpainful group was more affected by surgery with larger changes in Mobility and UE domains, perhaps due to their higher preoperative function. Understanding the etiology of preoperative pain is crucial for planning and patient counseling. Further studies are needed to monitor improvement over time.

## P52. A quick-scan of PROMIS® computerized adaptive tests supported the definition of patients with complex problems

### Leo D. Roorda, Simon Verberne

#### Amsterdam Rehabilitation Research Center | Reade, Amsterdam, Netherlands

##### **Correspondence:** Leo D. Roorda (leo.d.roorda.research@gmail.com)

**Background**

Multidisciplinary secondary care rehabilitation treatment is, as compared to monodisciplinary primary care treatments, an extensive and expensive treatment and, as a consequence, intended for patients with complex problems only. However, what are patients with complex problems? The aim of this study was to apply PROMIS® computerized adaptive tests (CATs) as a quick-scan to support the definition of patients with complex problems.

**Methods**

Patients with arthritis, referred to a multidisciplinary rehabilitation team of a secondary outpatient center for rehabilitation and rheumatology, were invited to participate. They completed, during the multidisciplinary team intake procedure, CAT-versions of the seven PROMIS profile domains, the PROMIS Upper Extremity domain, given the high prevalence of hand-related problem in patients with arthritis, and the PROMIS Satisfaction with Social Roles and Activities domain, given its relevance for rehabilitation medicine. PROMIS T-scores were calculated for which 50 represents the average score of the general Dutch (Anxiety and Depression) or US (other domains) population, with a SD of 10, and higher scores indicating more of the domain assessed. Patients with complex problems were defined as having ≥1 T-score indicating severe problems (≤30 for positively-worded domains, e.g., physical functioning, and ≥70 for negatively-worded domains, e.g., anxiety, respectively), ≥2 T-scores indicating moderate-to-severe problems (≤35 and ≥65, respectively), or ≥3 T-scores indicating moderate problems (≤40 and ≥60, respectively).

**Results**

A total of 102 persons (27.5% male; mean±SD age 48.8±15.7y., pain intensity [range 0-10] 6.0±1.8, fatigue [range 0-10] 7.4±1.9 and DAS-44 4.2±1.4) participated. Mean±SD PROMIS T-scores were: Physical Functioning, 35.7±6.5; Anxiety, 59.3±6.7; Depression, 55.2±9.6; Fatigue, 60.9±8.0; Sleep Disturbance, 56.0±9.5; Ability to Participate in Social Roles and Activities, 41.5±6.7; Pain Interference, 63.7±7.5; Upper Extremity, 30.9±6.0 and Satisfaction with Social Roles and Activities, 39.6±8.1. Eighty four out of 102 patients (82,4%) were categorized as complex.

**Conclusions**

A quick-scan with the PROMIS profile and two additional CATs supported the definition of patients with arthritis and complex problems. Future applications might be the use of PROMIS CATs to collaborate between primary and secondary care settings and, thus, supporting the creation of value for patients with healthcare networks.

## O53. Development, evaluation and use of item banks and CATs with older adults: a scoping review

### Rick Sawatzky^1,2^, Ayumi Sasaki^3^, Lara Russell^1,2^

#### ^1^Trinity Western University, Langley, British Columbia, Canada; ^2^Centre for Health Evaluation and Outcome Sciences, Vancouver, British Columbia, Canada; ^3^University of British Columbia, Vancouver, British Columbia, Canada

##### **Correspondence:** Rick Sawatzky (rick.sawatzky@twu.ca)

**Background**

The potential of computerized adaptive tests (CATs) to reduce respondent burden in quality of life assessments may be particularly beneficial to older adults who struggle with fatigue, reduced attention, and cognitive deficits. We conducted a scoping review to describe the extent to which CATs have been developed, evaluated and used with older adults.

**Methods**

Library databases (EMBASE, Medline, CINAHL, PsycInfo, and Web of Science Core Collection) were searched using the terms "computerized adaptive", "computer adaptive", or "computerised adaptive". References were imported into EndNote and duplicates removed. Initial screening of titles, abstracts and keywords was conducted to identify articles describing the development, evaluation or use of item banks or CATs in a health context. References were then filtered in EndNote using the term “Older adult OR elder OR senior OR geriatric OR gerontology”. The identified articles were subjected to a full-text review by two reviewers.

**Results**

The initial database search identified 6,795 articles. After removing duplicates and initial screening, 860 articles relating to the development, evaluation or use of item banks or CATs in health fields were retained. Filtering in EndNote resulted in 181 articles, of which 95 were retained after full-text review (86 did not focus on CATs or did not clearly include adults >60 years of age).

Only 23% of studies included a focus on older adults. Thirty-one percent used PROMIS measures, primarily the physical function, pain intensity and mental health item banks. Other measures appeared between 1 and 6 times. A number of studies (44%) reported on the development of item banks or CATs, and 69% primarily reported psychometric properties. Validity and/or reliability evidence was reported in about 45% of studies. Few studies reported on the use of CATs in research (e.g., as an outcome measure) or in clinical practice (16% and 1%, respectively).

**Conclusions**

Though there is some validity evidence for using CATs with older adults, very few studies focus on use in clinical practice. Further research that specifically focuses on routine clinical use of CATs for quality of life assessments with older adults is recommended.

## P54. Baseline results from the chronic kidney disease cohort of the NIH PEPR Consortium

### Julia Schuchard, Cortney Bruno, Sandra Amaral, Susan L. Furth, Christopher B. Forrest

#### Children’s Hospital of Philadelphia, Philadelphia, PA, USA

##### **Correspondence:** Julia Schuchard (schuchardj@email.chop.edu)

**Background**

Pediatric chronic kidney disease (CKD) is characterized by progressive decline in kidney function. This cross-sectional study aimed to evaluate the clinical validity of PROMIS person-reported outcome measures for children with CKD and advance the understanding of the impact of CKD on children’s quality of life.

**Methods**

We enrolled 212 dyads (patients with CKD ages 8-21 and a parent) from 15 pediatric nephrology clinics across the United States and one in Canada. Children completed six PROMIS pediatric measures (fatigue, sleep disturbance, sleep-related impairment, meaning and purpose, life satisfaction, and psychological experiences of stress). Parents completed four PROMIS parent proxy measures (positive affect, global health, anxiety, and depressive symptoms). Questionnaire data were linked to clinical information from electronic health records and cohort-specific case report forms. Variables significantly associated with a PROMIS measure in bivariate regression were included in a multiple regression model for the measure. Cohen's d scores indicate the standardized difference between mean PROMIS T-scores.

**Results**

Kidney function, as measured by estimated glomerular filtration rate, was not significantly associated with the PROMIS measures. Significant results included worse global health for children with short stature (Cohen’s d = 0.58) and worse sleep-related impairment (Cohen’s d = 0.71) and fatigue (Cohen’s d = 0.59) for children who had been hospitalized in the past three months. Of the clinical and sociodemographic variables investigated, the factor most consistently associated with worse PROMIS T-scores was parent-reported presence of a sleep problem (Cohen’s d greater than 0.4 for every PROMIS measure in the study). Worse child-reported sleep disturbance was also significantly associated with worse scores on every other child-reported PROMIS measure (Pearson r values 0.34 - 0.49) as well as parent proxy global health, anxiety, and depressive symptoms (Pearson r 0.22 – 0.27).

**Conclusions**

Results support the validity of PROMIS measures for assessing the physical and emotional experiences of children with CKD. Findings show that sleep problems are associated with a wide range of health-related quality of life outcomes in this population. Routinely asking about sleep problems in pediatric nephrology clinics may help identify targets of intervention to improve the lived experiences of children with CKD.

## O55. The effects of cognitive function on patient-reported outcomes in Parkinson disease

### Lisa M Shulman and Ann L Gruber-Baldini

#### University of Maryland School of Medicine Department of Neurology and Department of Epidemiology and Public Health, Baltimore, MD, USA

##### **Correspondence:** Lisa M Shulman (LShulman@som.umaryland.edu)

**Objective**

Reliable responses on patient-reported outcome measures (PROMs) depend upon intact memory and insight. This study investigates the relationship between cognitive function and patient-reported outcomes in Parkinson disease (PD).

**Methods**

296 PD patients were divided into 3 subgroups based on cognitive ratings on the Montreal Cognitive Assessment (MoCA): 1) Cognitively Intact (MoCA 26-30; N=160), 2) Mild Cognitive Impairment (MCI; MoCA 20-25; N=97), 3) Cognitively Impaired (MoCA 0-19; N=39). Differences in patient-reported outcomes (PROMIS-29 Profile, Self-Efficacy for Managing Conditions), and Clinician-reported outcomes (PD severity, disability) were examined by ANOVA. Multivariable regressions examined associations between cognitive status and other outcomes controlling for disease severity and demographics.

**Results**

The sample was mean age 68.5(9.7) years, 67% male, 86% white, 40% college educated, PD duration 6.6(6.1) years. The 3 cognitive subgroups differed in age, race, PD severity and disability, but not sex or education. Greatest disability was in the cognitively impaired group, least disability in the cognitively intact group, and intermediate disability in MCI. Significant differences between the 3 cognitive subgroups were shown on 4 out of 7 PROMIS-29 measures, and 5 out of 5 Self-Efficacy domains (PROMIS-29 physical function, anxiety, depression, social ability, but not fatigue, sleep or pain; PROMIS Self-Efficacy for Managing Daily Activities, Symptoms, Emotions, Medications, Social Interactions; p<.001). Greater cognitive impairment was consistently associated with greater physical/mental impairment, and less self-efficacy. Multivariable regressions, controlling for disease severity, showed PD severity was the strongest predictor of outcomes, but differences by cognitive subgroup remained significant for PROMIS Anxiety and Self-Efficacy for Managing Medications. Analysis of correlations between continuous MoCA data and Self-Efficacy domains, showed the highest correlation with Managing Medications (r=.53) and the lowest correlations with Managing Emotions and Social Interactions (r=.29).

**Conclusions**

Cognitive impairment in PD is associated with patient-reported impairment of physical function and mental health, and less self-efficacy for managing conditions. The largest effects of cognitive impairment were on anxiety and self-efficacy for managing medications. PD patients with cognitive impairment experienced greater anxiety and less confidence with managing medication regimens. The results of this study suggest that responses on PROM’s retain meaningful information in PD patients with cognitive impairment.

## P56. Self-efficacy as a predictor of outcomes

### Lisa M Shulman, and Ann L Gruber-Baldini

#### University of Maryland School of Medicine Department of Neurology and Department of Epidemiology and Public Health, Baltimore, MD, USA

##### **Correspondence:** Lisa M Shulman (LShulman@som.umaryland.edu)

**Objective**

To investigate self-efficacy (SE) for managing chronic conditions as a predictor of outcomes in Parkinson disease (PD), across the 5 PROMIS self-efficacy domains.

**Methods**

A sample of 293 PD patients were identified with one-year longitudinal data with the following assessments: 1) PROMIS Self-Efficacy for Managing Chronic Conditions, 2) PROMIS Profile-29 and Global Health, and 3) Unified Parkinson’s Disease Rating Scale (UPDRS). The 5 PROMIS SE domains are: Managing Daily Activities, Managing Symptoms, Managing Medications/Treatments, Managing Emotions and Managing Social Interactions. Multivariable regressions predicted 1-year outcomes (PROMIS-29: anxiety, depression, physical functioning, fatigue, sleep, social ability, pain), PROMIS Global Health and UPDRS) by the 5 SE domains, controlling for baseline characteristics.

**Results**

The sample was mean age 67.2(9.2) Y, 61% M, PD duration 6.6(5.8) Y. Baseline Self-Efficacy for Managing Daily Activities independently predicted the most outcomes after one-year including depression, fatigue, physical functioning, social activity, UPDRS Total and PROMIS Physical and Mental Global Health (p<.0001 to p<.05). SE for Managing Medications predicted anxiety, depression, fatigue and Mental Global Health. SE for Managing Emotions predicted pain intensity, sleep and UPDRS total, but no mental health outcomes. SE for Managing Symptoms predicted only pain intensity. SE for Managing Social Interactions was not an independent predictor of any outcome including ability to participate in social roles/activities. Only one outcome was not predicted by any SE domain- the UPDRS Motor Examination.

**Conclusions**

The 5 domains of PROMIS Self-Efficacy for Managing Chronic Conditions independently predict different disease outcomes. SE for Managing Daily Activities predicted the greatest number and range of outcomes including mental health, fatigue, disability, social activity and general PD severity. A dichotomy of outcomes prediction was found between the different domains: Managing Daily Activities and Medications (disability/mental health outcomes) vs. Managing Emotions and Symptoms (pain/sleep). Notably, the only outcome not predicted by baseline SE was the most objective outcome (motor function based on neurologic examination). This study confirms previous evidence that self-efficacy for managing chronic conditions is a potent predictor of disease outcomes. Data on the level of self-efficacy in different PROMIS domains can be used to guide management based on individual risk profiles.

## P57. The efficacy of using medical art therapy to intervene on the psychosocial well-being of cancer survivors

### Heather P. Tarleton^1^, Debra Linesch^2^, Einat S. Metzl^2^, Arash Asher^3^

#### ^1^ Department of Health and Human Sciences, Loyola Marymount University, Los Angeles, CA, USA; ^2^ Department of Marital and Family Therapy, Loyola Marymount University, Los Angeles, CA, USA; ^3^ The Samuel Oschin Comprehensive Cancer Institute, Cedars-Sinai Medical Center, Los Angeles, CA, USA

##### **Correspondence:** Heather P. Tarleton (Heather.Tarleton@lmu.edu)

**Background**

Oncologists indicate that poor mental health, emotional distress, and loneliness among cancer patients is of major concern. These psychosocial experiences might be managed through a medical art therapy intervention to promote mental health and psychosocial well-being of cancer survivors.

**Methods**

Two pilot studies of medical art therapy interventions (2018, 2019) were implemented with cancer survivors, with approval by the IRB at Loyola Marymount University. Groups were held once a week for 2-hours, for twelve weeks, and administered as an open-ended process group with art materials and art directives for ongoing expression of experiences as cancer survivors. Given that medical art therapy is psychotherapy, each group was implemented by a licensed clinical art therapist. Post-intervention interviews were held with five participants from the 2018 study. Each interview lasted from 45-60 minutes and transcripts were analyzed using NVivo software. Four participants from the 2019 study completed the following questionnaires at both baseline and post-intervention: DSM-5 Self-Rated, PROMIS-Global Health, Fatigue-Short form 8a, Facit Sp-EX, Edmonton Symptom Assessment System, Beck Hopelessness Scale, UCLA Version 3 Loneliness Scale, Beck Anxiety Inventory, Beck’s Depression Inventory.

**Results**

In the interview analysis, four thematic areas emerged – methods, outcomes, life as a cancer patient and suggestions for future research. Participants in the 2018 study developed new insights into their experience as cancer patients (e.g. gratitude, value of non-verbal communication, positive social connections). Two participants reported that the process helped with cognitive and communication issues that were side-effects of cancer medications and treatments. Participants in the 2019 study experienced decreases in severity of mental health and emotional problems reported at baseline. Participants indicated improvements in feeling more connected, particularly for the three who indicated some hopelessness or disconnection at baseline. Two participants who indicated that they were unsure, frightened about the future showed marked improvement in their optimism for the future. The assessment of loneliness showed the most consistent improvement across participants, and there was also a reported increase in meaningfulness in their relationships with others.

**Conclusion**

Overall this data, despite sample size limitations, does provide valuable information for the field. It suggests that the art therapy process is an impactful one, likely to be responsive to heightened research methods with larger numbers of participants. It also suggests that more complex domains with intertwined physical and mental/emotional components, like fatigue and anxiety, may be less responsive to an art therapy intervention as compared to depression, loneliness and spiritual well-being.

## O58. Psychometric assessment of the PROMIS Scale v1.2 Global Health in the general Dutch population: an item response theory analysis

### Leonardo Pellicciari^1^, Alessandro Chiarotto^2^, Emanuele Giusti^3^, Leo D. Roorda^4^, Caroline B. Terwee^5^

#### ^1^Department of Neurorehabilitation, IRCCS San Raffaele Pisana, Rome, Italy; ^2^Department of Health Sciences, Amsterdam Movement Sciences research institute, VU University, Amsterdam, the Netherlands; Department of General Practice, Erasmus MC, University Medical Center, Rotterdam, the Netherlands; ^3^IRCCS Istituto Auxologico Italiano, Psychology Research Laboratory, Oggebbio (VCO); Italy; Catholic University of the Sacred Heart, Department of Psychology, Milan, Italy; ^4^Amsterdam Rehabilitation Research Center | Reade, Amsterdam, the Netherlands; ^5^Amsterdam UMC, Vrije Universiteit Amsterdam, Department of Epidemiology and Biostatistics, Amsterdam Public Health research institute, Amsterdam, the Netherlands

##### **Correspondence:** Caroline B. Terwee (cb.terwee@amsterdamumc.nl)

**Background**

We aimed 1) To perform a psychometric assessment of the Patient-Reported Outcomes Measurement Information System (PROMIS®) Scale v1.2 Global Health (PROMIS-GH) in the general Dutch population; and 2) to study its cross-cultural validity comparing the Dutch and the United States (US) versions.

**Methods**

The 10-item PROMIS-GH was administered online to a sample representative of the Dutch general population. The psychometric properties of the 4-item Global Mental Health (GMH) and 4-item Global Physical Health (GPH) subscales were studied through analyses of dimensionality and local dependence (Confirmatory Factor Analysis), monotonicity (Mokken scale analysis), fit to a Graded Response Model, Item Response Theory parameters, and measurement invariance (through a Differential Item Functioning [DIF] analysis for age, gender, education, region, ethnicity). Cross-cultural validity was studied by evaluating DIF for language (Dutch versus English).

**Results**

The GMH and GPH were completed by 4370 people living in the Netherlands (years [mean±standard deviation]=51.3±16.6; 47.3% male). Unidimensionality was supported for GMH (CFI=0.984; TLI=0.952, RMSEA=0.219) and GPH (CFI=0.991; TLI=0.973; RMSEA=0.116) and no local dependence (all residual correlations<0.20) was found. Monotonicity (H=0.602 and 0.537 for GMH and GPH, respectively) was sufficient, and data fitted to the model (RMSEA=0.026 and 0.019 for GMH and GPH, respectively). However, all items exhibited misfit to the GRM model (S-X2 p values<0.0001). After adjusting for type-I error (by creating 10 random samples of 473 subjects), ten analyses showed satisfactory item fit statistics for all items (S-X2 p-values≥0.001) except for Global02, Global04, and Global05 (p<0.001 in only one analysis) and Global10r (p<0.001 in six analyses) for GMH, and Global07r (p<0.001 in only one analysis) for GPH. Global08r for GPH showed DIF for age (McFadden’s pseudo R2 change=0.05); cross-cultural validity was supported (no DIF for language).

**Conclusions**

GMH and GPH exhibited sufficient psychometric performance in the general Dutch population, and could be used to measure global health across different populations. However, the GMH could be improved as Global10r showed misfit to the GRM; moreover, it presented the lowest item-scale correlation, discrimination parameter and information value.

Further studies are recommended on clinical samples and in different countries, to better evaluate if a modification of the scale is necessary.

## O59. Validation of the Dutch-Flemish PROMIS® upper extremity v2.0 item bank in patients with upper extremity disorders

### Caroline B. Terwee^1^ , EJA Haan^1,2,3^, CM Lameijer^4^, SGJ van Bruggen^4^, MF Van Wier^5^, NW Willigenburg^5^, DFP Van Deurzen^5^ MF Pisters^2^, AJ Kaat^6^, V Stouten^7^, K Van der Elst^7,8^, LD Roorda^3^

#### ^1^Amsterdam UMC, Vrije Universiteit Amsterdam, Department of Epidemiology and Biostatistics, Amsterdam Public Health Research Institute, Boelelaan 1117, Amsterdam, The Netherlands; ^2^Physical Therapy Sciences, Program in Clinical Health Sciences, University Medical Center Utrecht, Utrecht University, The Netherlands; ^3^Amsterdam Rehabilitation Research Center | Reade, Amsterdam, The Netherlands; ^4^Amsterdam UMC, Vrije Universiteit Amsterdam, Department of Traumasurgery, Boelelaan 1117, Amsterdam, The Netherlands; ^5^Department of Orthopedic Surgery, Joint Research, OLVG, Amsterdam, The Netherlands; ^6^Department of Medical Social Sciences, Feinberg School of Medicine, Northwestern University, Chicago, IL, USA; ^7^KU Leuven - University of Leuven Department of Development and Regeneration, Skeletal Biology and Engineering Research Center, Leuven, Belgium; ^8^University Hospitals Leuven, Department of Rheumatology, Leuven, Belgium

##### **Correspondence:** Caroline B. Terwee (cb.terwee@amsterdamumc.nl)

**Background**

The 46-item PROMIS® Upper Extremity (UE) item bank v2.0 was developed to replace the 16-item v1.2 version, to increase the measurement range and improve the psychometric properties. We aimed to evaluate the psychometric properties of the Dutch-Flemish PROMIS☐ UE item bank v2.0 in patients with upper-extremity disorders.

**Methods**

The full item bank was completed by 521 patients with upper extremity disorders from two trauma centers. Assumptions of the IRT model (unidimensionality, local independence, and monotonicity) were checked and a Graded Response Model was fitted. Item fit and item parameters were estimated. The number of patients for whom a theta could be estimated with SE<0.32 (reliability>0.90) was calculated, based on the full item bank, the standard 7a short form (SF7a), and a simulated CAT, and compared to the 30-item DASH and 17-item MHQ-ADL scale. Differential Item Functioning (DIF) was examined for age, gender, duration of complaints (<6 months versus ≥6 months, location of complaints (hand/wrist versus arm/shoulder) center, and language (Dutch versus English, comparing to a US sample of 246 patients with upper extremity pain or function).

**Results**

The assumptions for IRT were considered met (CFI: 0.93, TLI: 0.93, RMSEA: 0.10, SRMR: 0.09, ratio 1th/2nd factor: 10.7, Omega H 0.80, ECV 0.68) and all items fitted the GRM model. A theta could be estimated with SE<0.32 for 97.1% of the population when using the full item bank, for 39.0% when using SF7a, and for 92.7% when using CAT (average 4.8 items). Reliability of the full item bank was better than the DASH and MHQ-ADL. The DASH and MHQ-ADL had better reliability than the SF7a and CAT, but required more items to complete. One item was flagged for gender DIF, 3 for duration of complaints DIF, 14 for location of complaints DIF, 7 for center DIF, and 3 for language DIF. The impact of DIF on T-scores was minimal.

**Conclusions**

This is the first validation study of the PROMIS☐ UE item bank v2.0 outside of the US. The Dutch-Flemish PROMIS item bank Upper Extremity v2.0 showed sufficient psychometric properties in a Dutch population with upper extremity disorder.

## O60. TBI-QOL composite scores for measuring global and domain-specific quality of life

### Callie Tyner^1^; David Tulsky^1^; Aaron Boulton^1^; Pamela Kisala^1^; Joseph Glutting^2^; Mark Sherer^3^

#### ^1^Center for Health Assessment Research and Translation, University of Delaware, Newark, DE, USA; ^2^University of Delaware School of Education, Newark, DE, USA; ^3^TIRR Memorial Hermann, Houston, TX, USA

##### **Correspondence:** Callie Tyner (ctyner@udel.edu)

**Background/Objective**

The Traumatic Brain Injury Quality of Life Measurement System (TBI-QOL) was developed to optimize and extend PROMIS® and Neuro-QoL for traumatic brain injury (TBI). The TBI-QOL includes 5 PROMIS® items banks recalibrated for TBI and transformed to the PROMIS® metric, 9 Neuro-QoL item banks recalibrated for TBI and transformed to the Neuro-QoL metric, and 6 novel item banks developed using the PROMIS® methodology and calibrated on a TBI-specific metric. The 20 TBI-QOL item banks measure aspects of physical, emotional, cognitive, and social domains; each item bank is scored and interpreted independently. This can pose a challenge for clinicians and researchers wishing to measure post-TBI health outcomes from the patient’s perspective, given the number of item banks that need to be interpreted simultaneously. The objective of this project was to develop composite scores to increase parsimony of interpreting global and domain-specific outcomes post-TBI.

**Methods**

TBI-QOL item banks were administered to 504 community-dwelling individuals, ages 18-64, with a medical-record-confirmed history of TBI. Five composite scores were computed from 9 item banks using a nonlinear area conversion/normalized transformation method: Global QOL, Physical Health, Emotional Health, Cognitive Health, and Social Health. The 9 item banks that were selected for inclusion were all optimized PROMIS® or Neuro-QoL item banks. Each composite index score was created to reflect a normal distribution (M = 100; SD = 15).

**Results**

The resulting composite scores share a uniform direction of interpretation, with higher scores indicating better functioning. Published confidence intervals aid interpretation. Correlations between item banks and the corresponding domain composites were all > .50. The highest correlations with the Global composite were cognition, social functioning, mood, and fatigue. No systematic differences in composite scores were detected by age or injury severity.

**Conclusions**

These composite scores offer a reliable method for measuring global and domain-specific QOL after TBI using only 9 PROMIS®/Neuro-QoL item banks. Composite scores can be computed using T-scores from the component item banks from computer adaptive tests or fixed-length short forms. Future research could take this approach for other PROMIS® item banks and domains to provide users with parsimonious estimates of QOL.

## P61. Face and content validity of the Anterior Cruciate Ligament Quality of Life Questionnaire (ACL-QOL) sport domain items

### Jeremy Tynedal^1^, Mark Lafave^2^, S. Mark Heard^1,3^, Greg Buchko^1^, Laurie Hiemstra^1,3^, Michaela Kopka^1^, Sarah Kerslake^1^

#### ^1^Banff Sport Medicine, Banff, Alberta, Canada; ^2^Mount Royal University, Calgary, Alberta, Canada; ^3^University of Calgary, Calgary, Alberta, Canada

##### **Correspondence:** Jeremy Tynedal (research@banffsportmed.ca)

**Background**

Patient-reported outcome measures (PROMs) enable patients with anterior cruciate ligament reconstruction (ACL-R) to contribute an assessment of their outcome to the evaluation of treatment. Unfortunately, most ACL-R PROMs lack positive evidence in areas of the Consensus based standards for the Selection of health Measurement Instruments (COSMIN). The ACL-Quality of Life questionnaire (ACL-QOL) is a PROM that fulfills 8/9 COSMIN criteria across 5 domains. Sport/recreation is the largest and most frequently uncompleted domain. This may limit the utility of the ACL-QOL by patients and clinicians.

**Methods**

A mixed method design was used to nest quantitative data within qualitative data. 92 ACL-R patients and 5 clinicians participated in survey and open-ended interview questions. Participants rated the relevance of the 12 sport-domain items and 3 pilot items out of 10, and identified the 6 most important items. Patients indicated whether they played a contact sport. Participants identified any readability challenges, redundant items and concepts not covered in the sport-domain.

Paired t-tests of mean item ratings were used to analyze differences between patient groups by age-range or sex. Rating scores and item frequencies were compared between patients and clinicians. Readability of each sport item was assessed using the https://readability.io tool. 3 independent assessors completed content analysis by clustering codes into categories and debriefing to compare themes.

**Results**

Patients were mean age 34 years, mean 13 months post-operative ACL-R and 56.5% male. There were no statistically significant differences in mean item scores between patient groups. The same 6 items were endorsed by patients and clinicians. 16% of patients participated in contact sport. 5/12 items were assessed above a 10^th^ grade reading level. 21% of patients identified readability challenges, 54% redundant items, and 43% missing concepts.

There were descriptive differences in item ratings between patient groups. Females rated items consistently lower than males. Increased representation of recreation/non-recreational activities and a greater risk appraisal for sport resumption were issues mostly raised by older patients.

**Conclusions**

ACL-R PROMs should contain gender-neutral language, simpler words and decreased emphasis on organized/competitive sport. Factor analysis and item reduction of the ACL-QOL could create a more accurate, user-friendly PROM and decrease the cost-burden of utilization.

## P62. Withdrawn

## P63. Withdrawn

## O64. Validation of PROMIS Profile-29 in adults with Haemophilia in the Netherlands

### Erna C. van Balen^1^, Lotte Haverman^2^, Shermarke Hassan^1^, Liesbeth M. Taal^1^, Cees Smit^1^, Mariëtte H. Driessens^3^, Erik A.M. Beckers^4^, Michiel Coppens^5^, Jeroen C.J. Eikenboom^6^, Hélène L. Hooimeijer^7^, Frank W.G. Leebeek^8^, Evelien P. Mauser-Bunschoten^9^, Lize F.D. van Vulpen^9^, Saskia E. M. Schols^10^, Caroline B. Terwee^11^, Frits R. Rosendaal^1^, Johanna G. van der Bom^1,12^, Samantha C. Gouw^1,13^

#### ^1^ Department of Clinical Epidemiology, Leiden University Medical Center, The Netherlands; ^2^ Psychosocial Department, Amsterdam UMC, University of Amsterdam, Amsterdam, The Netherlands; ^3^ Dutch Society of Haemophilia Patients (NVHP), Nijkerk, The Netherlands; ^4^ Department of Hematology, Maastricht University Medical Centre, Maastricht, The Netherlands; ^5^ Amsterdam Cardiovascular Sciences, Department of Vascular Medicine, Amsterdam UMC, University of Amsterdam, The Netherlands; ^6^ Department of Internal Medicine, division of Thrombosis and Hemostasis, Leiden University Medical Center, Leiden, The Netherlands; ^7^ Department of Paediatrics, University Medical Center Groningen, Groningen, The Netherlands; ^8^ Department of Hematology, Erasmus University Medical Center, Rotterdam, The Netherlands; ^9^ Van Creveldkliniek, University Medical Center Utrecht, Utrecht, The Netherlands; ^10^ Department of Hematology, Radboud University Medical Center, Nijmegen, The Netherlands and Hemophilia Treatment Center Nijmegen-Eindhoven-Maastricht, The Netherlands; ^11^ Department of Epidemiology and Biostatistics, Amsterdam Public Health Research Institute, Amsterdam UMC, Vrije Universiteit Amsterdam, The Netherlands; ^12^ Center for Clinical Transfusion Research, Sanquin Research, Leiden, The Netherlands; ^13^ Department of Pediatric Hematology, Emma Children's Hospital, Amsterdam UMC, University of Amsterdam, The Netherlands

##### **Correspondence:** Erna C. van Balen (e.c.van_balen@lumc.nl)

**Background**

Assessing health-related quality of life (HR-QoL) is increasingly important in the congenital bleeding disorder haemophilia, which has evolved from a fatal to a chronic condition with a near-normal life expectancy. HRQoL in this population is mostly measured with PROMs based on Classical Test Theory, such as the RAND-36. PROMIS item banks measure HRQoL more comprehensively and precisely compared to legacy questionnaires, but they need to be validated in specific patient groups. Therefore, the aim of this study was to validate the PROMIS® v2.01 Profile-29 in adults with haemophilia.

**Materials and methods**

All Dutch men, participating in the ongoing sixth ‘Haemophilia in the Netherlands’ study, completed electronic or paper questionnaires that consisted of the RAND-36 and PROMIS Profile-29, and socio-economic and clinical characteristics. Construct validity was investigated by assessing convergent, discriminative and structural validity. Convergent validity of PROMIS Profile-29 was assessed by calculating Pearson correlation coefficients between PROMIS T-scores and corresponding sum scores of RAND-36 measuring similar constructs. Correlations were expected to be strong (≥0.80) between PROMIS Profile-29 and RAND-36 domains. Discriminative validity was assessed with known-groups analysis of different clinically relevant severity levels of haemophilia, with expected worse scores for those with severe haemophilia.

**Results**

Of 730 patients who completed the questionnaires, 379 had mild, 92 had moderate and 256 had severe haemophilia. Pearson’s r’s were 0.91 for PROMIS and RAND-36 physical functioning, 0.74 for PROMIS anxiety and depression and RAND-36 mental health, 0.70 for PROMIS ability to participate in social roles / activities and RAND-36 social functioning, 0.82 for PROMIS pain interference and RAND-36 pain, and 0.80 for PROMIS pain intensity and RAND-36 pain. Mean PROMIS T-scores were worse for severe compared to mild haemophilia for all PROMIS Profile-29 domains: 43.7 and 52.1 for physical functioning, 48.4 and 47.9 for anxiety, 47.5 and 45.9 for depression, 47.6 and 45.9 for fatigue, 47.1 and 45.8 for sleep disturbance, 51.6 and 55.4 for ability to participate in social roles, and 52.9 and 47.5 for pain interference.

**Conclusions**

In this preliminary analysis of approximately 50 percent of the total Dutch haemophilia population, construct validity of all PROMIS Profile-29 constructs was reasonable. Differences between subgroups were as expected.

## P65. Cross-specialty PROMIS-10 differential item functioning

### Paul M Werth^1^, Clifford A Reilly^1^, David S Jevsevar^1,2^

#### ^**1**^ Department of Orthopaedics, Dartmouth-Hitchcock Medical Center, Lebanon, NH, USA; ^2^Department of Orthopaedics, Dartmouth Geisel School of Medicine, Hanover, NH, USA

##### **Correspondence:** Paul M Werth (paul.m.werth@hitchcock.org)

**Background**

The various PROMIS measures have been tested for invariance across age, gender, socioeconomic status, and cross-culturally [1-4]. Generally, the items function well as demonstrated by uniformity of discriminative and difficulty parameters across groups. We hypothesize that the PROMIS Global-10 will perform similarly when compared across a variety of medical specialties. To this point, we sought to determine if aspects of the clinical context in which the PROMIS Global-10 measure was administered affected item functioning. Specifically, this study focuses on the functioning of the item grouping associated with the physical health (PCS) and mental health (MCS) domains across these groups. To our knowledge, no study demonstrates the lack of differential item functioning (DIF) for the PROMIS Global-10 across specialties.

**Materials and methods**

6570 complete PROMIS Global-10 measures were retrospectively analyzed using the ‘mirt’ packaged on the R platform across three medical specialties (*N*_group_ = 2190). Unidimensional multi-group 2PL graded response models were analyzed for both MCS and PCS with the general item (i.e., Global item 1) serving as the anchor for both analyses. DIF was investigated for both MCS and PCS in patients from Orthopaedic Surgery, Family Medicine, and Internal Medicine using quasi Monte Carlo estimation. To assess the significance of DIF, Wald tests were used with the Benjamini & Hochberg procedure [5].

**Results**

All, but the last item were resilient to DIF across medical specialty. Global item 10 did demonstrate statistically significant DIF (Wald: 6.51, *p* = .04).

**Conclusions**

The results suggest that items associated with the MCS and PCS function well across medical specialty. Though the last item demonstrated statistically significant DIF, its clinical significance is in question considering the uniformity represented by the trace line plot. Of note, the plot demonstrates that the response options have a right skew on the latent construct.

**Ethics Approval**

Study approved by Dartmouth College by Institutions Ethnics Board, approval number STUDY00031786.

## P66. The development of a phase-specific patient-reported outcomes measurement system for patients with breast cancer

### Changrong Yuan, Qingmei Huang, Fulei Wu , Wen Zhang, Lei Cheng

#### School of Nursing, Fudan University, Shanghai, China

##### **Correspondence:** Changrong Yuan (yuancr@fudan.edu.cn)

**Objectives**

Patients with breast cancer (BCPs) often deal with different core health distresses, which may deeply influence the quality of life of patients as well as the recovery from the aspects of physical, physiological, and social health. The systematic collection of patient-reported outcomes (PROs) could identify the health distresses of patients so that potentially improve the quality of life of patients. This study focused on postoperative BCPs and BCPs receiving chemotherapy, and aimed to develop a treatment phase-specific Patient-reported Outcomes Measurement System-Breast Cancer (PROMS-BC), in order to provide a systematic and comprehensive assessment and evaluation system to promote the use of PROs in Chinese BCPs.

**Methods**

The conceptual framework of PROMS-BC was developed and identified by qualitative interview of BCPs and Delphi expert consultation. And then according to the methodology used in development of PROMIS® instruments, the development of treatment phase-specific breast cancer outcomes measures included systematic literature review, item evaluation, classification, and screening, cognitive review, and expert review. Finally, classic test theory (CTT) and item response theory (IRT), item analysis, item-total correlation, exploratory factor analysis, Cronbach’s α coefficient, and graded response model (GRM) were used to evaluate the measurement property of each item to decide the final inclusion of items.

**Results**

13 domains of PROMS-BC-Surgery and 18 domains of PROMS-BC-Chemotherapy were determined respectively. The CTT-based item evaluation showed that most of the items have good discrimination; The item-dimensions/ total scores correlation coefficients were satisfying except for some items showing strong correlation (r<0.6, p<0.01) with the dimension that was not theoretically belonged to; the factor structures and factor loadings were acceptable while few items showing double factor loadings; Corrected item total correlation of few item was<0.5 and the Cronbach α coefficient significantly improved after the item deleted. The IRT-based item evaluation suggested that most items performed well in discrimination and difficulty parameter, item characteristic curves (ICC) and scale information functions (SIF) were ideally distributed.

**Conclusions**

PROMS-BC is able to specifically reflect health distresses of postoperative BCPs and BCPs receiving chemotherapy, which helps to identify the physical, psychological and social health status of patients comprehensively.

## O67. Patient-reported symptom burden and function outcomes for breast cancer patients receiving chemotherapy based on latent profile analysis

### Changrong Yuan, Qingmei Huang, Wen Zhang, Lei Cheng

#### School of Nursing, Fudan University, Shanghai, China

##### **Correspondence:** Changrong Yuan (yuancr@fudan.edu.cn)

**Objective**

Women who are receiving chemotherapy for breast cancer often experience multiple, concurrent, troubling symptom which puts a heavy burden on patients and deteriorate their functions. This study was aimed to evaluate symptom severity and group patients with different profiles of symptom burden, and compare different function outcomes of breast cancer patients with different profiles.

**Methods**

Cross-sectional study was conducted and the treatment phase-specific Patient-Reported Outcomes Measurement System-Breast Cancer (PROMS-BC) developed by professor Yuan Changrong based on the methodology of PROMIS®, were used to evaluate the symptom burden for breast cancer patients who are during thermotherapy treatment. Latent profile analysis (LPA) were performed to determine the patient subgroups with different profiles of symptom burden.

**Results**

246 eligible patients were included and five most-common symptom including sleep disturbance, pain, fatigue, anxiety, depression, and body image for breast cancer patients during chemotherapy were evaluated by PROMS-BC measures. Three latent profiles were identified by LPA. 48 patients (19.5%) in the profile of “all high” experienced high level of all the above symptom burden, 74 patients (30.1%) who experienced low level of all the five symptom burden were in the subgroup of “all low”, in addition, about half of the patients (n=124, 50.4%) were in the profile of “moderate” symptom burden. Patients in the “all high” subgroup had the worst physical function status, a significantly lower cognitive function and a poorer social activities participation abilities.

**Conclusions**

LPA revealed that women who receive the same treatment can experience very different symptom burdens. Future research need to examine the characteristic of the patients in the profile of “all high” symptom burden in order to make clinicians focus their care on individuals most in need of symptom management and support.

## P68. Chinese clinicians’ perceptions and intentions towards the use of patient-reported outcomes: a preliminary investigation

### Fulei Wu^1^, Changrong Yuan^2^, Doris Howell^3^, Yang Yang^4^, Yingting Zhang^2^, Huan Liu^2^, Wen Zhang^2^

#### ^1^School of Nursing, Second Military Medical University, Shanghai, China; ^2^School of Nursing, Fudan University, Shanghai, China; ^3^Department of Supportive Care, Princess Margaret Cancer Centre, Toronto, Canada; ^4^Department of Medical Oncology, Oncology Hospital Affiliated to Fudan University, Shanghai, China

##### **Correspondence:** Wen Zhang (zhangwenivy@aliyun.com)

**Background**

Patient-reported outcomes (PROs) have shown benefits for improving patients experience, promoting patient-health professional communication, and health care performance when integrated into clinical practice. However, as the core stakeholder, the attitude of clinicians, specifically doctors and nurses, towards PROs have not been fully understood in China. This study aimed to explore the awareness, perceptions, and intention of using PROs in Chinese clinicians to provide evidence for building a clinical implementation of PROs in our future study.

**Methods**

A total of 591 participants were recruited by convenience sampling. A self-designed questionnaire with 8 questions of 0-4 Likert response and an open inquiry was used to investigate the awareness, perception, and intention of using PROs. Mean (standard deviation) and frequency were used for descriptive statistics. Univariate and multivariate logistic regression and linear regression were applied to identify the influencing factors. Data from the open question was analyzed by content analysis.

**Results**

The awareness rate of PROs was 64.6% in total. The mean score of perception and intention of using PROs were 2.41(0.74) and 2.19 (0.70) out of 4 respectively. The awareness was influenced by years of working, specialty, and the experience of training aboard. Participants who were nurses and had prior knowledge about PROs tended to have a higher level of perception on PROs. Participants with more years of working, who had prior knowledge about PROs and had a higher level of perception on PROs were more willing to integrate PROs in their future work. Information contributed by qualitative data include positive perceptions, negative perceptions, perceived knowledge gaps, and perceived support gaps.

**Conclusions**

The awareness, perception, and intention of using PROs in Chinese clinicians were at the medium level and were mainly influenced by clinicians’ previous knowledge and experience of PROs. A targeted educational and training program inclusive of the added clinical value of PROs, the interpretation of PROs results, and the professional feedback towards PROs results will be developed in our future study.

## P69. Identification of pain profiles in children and adolescents with cancer

### Wen Zhang^1^, Changrong Yuan^1^, Jiashu Wang^2^, Qingmei Huang^1^, Lei Cheng^1^

#### ^1^School of Nursing, Fudan University, Shanghai, China; ^2^School of Nursing and Health Management, Shanghai University of Medicine and Health Sciences, Shanghai, China

##### **Correspondence:** Wen Zhang (zhangwenivy@aliyun.com)

**Background**

This study was to group children and adolescents aged 5 to 17 with cancer by clusters of pain intensity, pain duration, pain interference and pain control by latent profile analysis (LPA), and to evaluate how these subgroups differed on demographic and Quality of Life-related outcomes.

**Methods**

275 children and adolescents aged 5 to 17 with cancer, from 5 tertiary hospitals in Shanghai and Suzhou, China were included in this study. Pain intensity, pain duration, pain interference and pain control were assessed by the Chinese version of compound self-reported pain assessment system in children and adolescents with cancer (translated from Pain Squad from Canada). QoL-related outcomes were measured by seven short forms of Pediatric PROMIS, including depress symptoms, anger, anxiety, fatigue, peer relationship, mobility, and upper extremity. Latent profile analysis (LPA) was used to identify latent classes of pain profiles.

**Results**

Four distinct pain classes were identified, including High (12.4%), Continuous (15.5%), Moderate (16.7%), and Low (55.3%). Guardian's employment (χ2=13.430, p=0.037), family monthly income (χ2=30.052, p=0.003), patient disease type (χ2=24.386, p=0.018), and outpatient or inpatient (χ2=18.227, p<0.001) were proved to have impact on patients’ pain profiles. The proportion of patients with neuroblastoma was much higher in the High class; and patients in the Continuous class more likely had unemployed guardians, less likely had high family monthly income (>RMB 3000), and more were inpatient. Compared to the Low class, patients in the High pain profile reported poorer mobility and upper extremity functions.

**Conclusions**

Four distinct pain profiles in children and adolescents with cancer were identified by LPA, assisting clinical staff to understand heterogeneity in pain patterns among different patients and their risk factors. By these findings, high risk patients (High and Continuous class) can be targeted. And significant relationships were found between pain profiles and some QoL-related outcomes, so patients in the High pain profile can be given more tailored intervention.

## O70. Do patient reported outcomes correlate with clinical findings in patients undergoing total knee arthroplasty?

### Parisa Ziarati^1^, Meredith L. Grogan Moore^2^, Adriana P. Lucas^1^, Paul M Werth^1^, Karl Koenig^2^, David S. Jevsevar^1^

#### ^1^Department of Orthopaedics, Dartmouth-Hitchcock Medical Center, Lebanon, NH, USA; ^2^Department of Surgery & Perioperative Care, Dell Medical School, University of Texas at Austin, Austin, TX, USA

##### **Correspondence:** Parisa Ziarati (parisa.ziarati.med@dartmouth.edu)

**Background**

The appropriate utilization rate of total knee arthroplasties (TKAs) is debated, and some suggest the rise in TKAs could be partially due to the subjective criteria used to identify patients for the procedure. Currently, the standard of care for assessing a potential TKA candidate includes using objective clinical findings such as Kellgren-Lawrence (KL) radiographic

scores and range of motion (ROM), preferably matched with patient reported outcome measures (PROMs). Although patient reported outcome PROMs are not currently used to adjust physician reimbursement, it is likely they will soon be employed in value-based payment reform as a driver of reimbursement. We sought to investigate whether PROMs correlate with clinical findings.

**Methods**

For 2266 patients that underwent a TKA procedure between 2012 and 2019, PRO scores and clinical measurements from the same pre-operative appointment (up to 90 days prior to surgery) were collected. Points of interest included knee KL grade, Patient-Reported Outcomes Measurement Information System (PROMIS)-10 Global subscores for Physical and

Mental Health (PROMIS-10 PH/MH), and knee ROM (degrees of flexion, extension) scores. Data was analyzed using R v.3.5.3. Spearman Rho Correlation analysis to determine the relationships between PROMIS-10 PM/MH and the clinical measurements of KL grade and ROM Flexion/Extension.

**Results**

The results demonstrate a small negative correlation between PROMIS-10 PH and KL score (*r* -0.070, p < 0.001), PROMIS-10 MH and KL score (r -0.040, p < 0.05), and PROMIS-10 PH and Extension score (*r* -0.051, p < 0.05). There was no statistically significant correlation between PROMIS-10 MH and Extension score, but the results did demonstrate a small positive correlation between PROMIS-10 PH and ROM Flexion score (*r* 0.306, p < 0.001), and PROMIS-10 MH and Flexion score (r 0.185, p < 0.001). These results suggest minimal, if any, correlation between the variables of interest.

**Conclusions**

PROMIS-10 PH and PROMIS-10 MH were not found to correlate strongly with clinical findings of radiographic severity or knee mobility in preoperative patients scheduled for TKA. These findings suggest a need for caution when establishing reliable TKA appropriateness criteria, especially with respect to using PROMs as a standalone assessment measure.

## P71. Recommended outcome domains for routine clinical care in chronic pain management: the patients’ perspective

### Diana Zidarov ^1, 2,3^, Alexia Zidarova-Carrié^4^, Sara Ahmed ^1,2,5^

#### ^1^ Faculty of Medicine, School of Physical and Occupational Therapy, McGill University, 3654 Prom. Sir William Osler, Montréal, Québec, Canada; ^2^ Centre de Recherche Interdisciplinaire en Réadaptation du Montréal Métropolitain, Montréal, Québec, Canada; ^3^ Institut universitaire sur la réadaptation en déficience physique de Montréal, Montreal, Quebec, Canada; ^4^Faculty of Medicine, Université Laval, Québec, Canada; ^5^ McGill Integrated University Health Network (RUIS) Centre of Expertise in Chronic Pain, Montréal, Québec, Canada

##### **Correspondence:** Diana Zidarov (diana.zidarov@umontreal.ca)

**Background**

Ten percent of the world’s population is affected by chronic pain (CP). To be able to develop a coordinated and effective patient management tailored to the specific needs of the person with CP, a comprehensive and appropriate clinical assessment is required. Deciding on what to measure in clinical practice must include the voice of patients to ensure outcomes reflect what is important to individuals with CP. The objective of this study was to identify the most important domains of health-related quality of life (HRQoL) affected by CP from the perspective of individuals suffering from CP.

**Methods**

An electronic cross-sectional survey was conducted with 64 patients with CP between November 2017 and August 2018 in Canada. The survey included: 1) the Patient Generated Index, an individualised measure of quality of life and 2) list of HRQoL domains from the Patient-Reported Outcomes Measurement Information System (PROMIS) framework to be ranked by importance.

**Results**

Patients nominated a total of 318 areas affected by CP. The most important areas in a person’s life affected by CP for which he/she would like to improve were *Recreation and leisure* (e.g. participating in social and family activities) (n=90; representing 25% of all nominated areas); *Global mental functions* (e.g. difficulty with sleep; self-esteem) (n= 45, representing 17% of all nominated areas); *Work and employment* (n=38; representing 15.5% of all nominated areas); *Walking and moving* (e.g. taking walks) (n=25; representing 9% of all nominated areas) and *Household tasks* (e.g. doing chores) (n=37; representing 8% of all nominated areas). In total, these areas represented 74% of all nominated areas. The five most important domains ranked by importance from the PROMIS framework were: *pain interference*, *pain intensity*, *sleep disturbance* and *physical function*, *fatigue* and *anxiety*.

**Conclusions**

These domains provide the most valued and relevant domains to be improved in settings offering multidisciplinary care to individuals with CP from the patient perspective. The results can be used in clinical care for joint decision-making and treatment planning to focus interventions on the areas of life most affected by CP and to identify appropriate patient-reported outcome measures to assess the outcomes of multidisciplinary interventions.

## P72. Examining predictors of achieving MCID following ACL reconstruction: the importance of preoperative PROMIS CAT scores

### Caleb M. Gulledge, Michael A. Korn, Sreten Franovic, Joshua Hester, Noah A. Kuhlmann, Vasilios Moutzouros, Eric C. Makhni

#### Henry Ford Health System, Detroit, MI, USA

##### **Correspondence:** Michael A. Korn (michael.korn10@gmail.com)

**Objective**

The primary purpose of this study was to determine if preoperative Patient-Reported Outcomes Measurement Information System (PROMIS) scores and patient-centric factors (PCF), as well as meniscal tears, impact the likelihood of achieving the minimal clinically important difference (MCID) after anterior cruciate ligament (ACL) reconstruction.

**Methods**

Patients who underwent ACL reconstruction between July 11, 2017 and October 3, 2018 and completed the PROMIS Physical Function (“PROMIS-PF”), PROMIS Pain Interference (“PROMIS-PI”), and PROMIS Depression (“PROMIS-D”) Computer Adaptive Tests (CAT) preoperatively and at two postoperative visits (3-months and 6-months) were included in this study. MCID was calculated using a distribution-based method, equal to one-half the standard deviation of preoperative scores. Predictive ability of preoperative and PCF were determined using a receiver operator characteristic curve utilizing the area under the curve.

**Results**

The mean preoperative PROMIS scores were 38.1 for PROMIS-PF, 60.3 for PROMIS-PI, and 47.6 for PROMIS-D, while the mean 6-month scores were 50.6, 49.4, and 41.1, respectively (p< 0.001). The proportion of patients achieving MCID at 6-months for PROMIS-PF was 86%, for PROMIS-PI was 75%, and for PROMIS-D was 55%. Preoperative cutoff values to predict not achieving MCID with 95% specificity at 6-months were ≥47.8 for PROMIS-PF, ≤52.7 for PROMIS-PI, and ≤39.5 for PROMIS-D. The time to surgery was found to predict the likelihood of achieving MCID for PROMIS-PF and PROMIS-PI, such that those with less time between injury and surgery were more likely to achieve MCID. However, all other PCF were not predictive of achieving MCID.

**Conclusions**

Preoperative PROMIS scores and the time from injury to surgery were found to predict the likelihood of achieving MCID after ACL reconstruction. Our findings suggest that preoperative PROMIS scores may have prognostic use in patients with ACL tears, and that reduced time to surgery may improve patient-reported outcomes.

